# DNAJC14-Independent Replication of the Atypical Porcine Pestivirus

**DOI:** 10.1128/jvi.01980-21

**Published:** 2022-07-19

**Authors:** Carina M. Reuscher, Kerstin Seitz, Lukas Schwarz, Francesco Geranio, Olaf Isken, Martin Raigel, Theresa Huber, Sandra Barth, Christiane Riedel, Anette Netsch, Katharina Zimmer, Till Rümenapf, Norbert Tautz, Benjamin Lamp

**Affiliations:** a Institute of Virology, Justus-Liebig-Universität Gießen, Giessen, Germany; b Institute of Virology, Department for Pathobiology, University of Veterinary Medicine, Vienna, Austria; c University Clinic for Swine, Department for Farm Animals and Veterinary Public Health, University of Veterinary Medicine, Vienna, Austria; d Institute for Virology and Cell Biology, University of Luebeck, Lübeck, Germany; Cornell University

**Keywords:** APPV, *Atypical porcine pestivirus*, *Pestivirus K*, monoclonal antibody, reverse genetics, DNAJC14, Jiv, cytopathogenicity, pestiviral biotype, NS3, NS2-3, pestivirus, biotype, cytopathogenic effect, monoclonal antibodies, reverse genetic analysis

## Abstract

Atypical porcine pestiviruses (APPV; *Pestivirus K*) are a recently discovered, very divergent species of the genus *Pestivirus* within the family *Flaviviridae*. The presence of APPV in piglet-producing farms is associated with the occurrence of so-called “shaking piglets,” suffering from mild to severe congenital tremor type A-II. Previous studies showed that the cellular protein DNAJC14 is an essential cofactor of the NS2 autoprotease of all classical pestiviruses. Consequently, genetically engineered DNAJC14 knockout cell lines were resistant to all tested noncytopathogenic (non-cp) pestiviruses. Surprisingly, we found that the non-cp APPV can replicate in these cells in the absence of DNAJC14, suggesting a divergent mechanism of polyprotein processing. A complete laboratory system for the study of APPV was established to learn more about the replication of this unusual virus. The inactivation of the APPV NS2 autoprotease using reverse genetics resulted in nonreplicative genomes. To further investigate whether a regulation of the NS2-3 cleavage is also existing in APPV, we constructed synthetic viral genomes with deletions and duplications leading to the NS2 independent release of mature NS3. As observed with other pestiviruses, the increase of mature NS3 resulted in elevated viral RNA replication levels and increased protein expression. Our data suggest that APPV exhibit a divergent mechanism for the regulation of the NS2 autoprotease activity most likely utilizing a different cellular protein for the adjustment of replication levels.

**IMPORTANCE** DNAJC14 is an essential cofactor of the pestiviral NS2 autoprotease, limiting replication to tolerable levels as a prerequisite for the noncytopathogenic biotype of pestiviruses. Surprisingly, we found that the atypical porcine pestivirus (APPV) is able to replicate in the absence of DNAJC14. We further investigated the NS2-3 processing of APPV using a molecular clone, monoclonal antibodies, and DNAJC14 knockout cells. We identified two potential active site residues of the NS2 autoprotease and could demonstrate that the release of NS3 by the NS2 autoprotease is essential for APPV replication. Defective interfering genomes and viral genomes with duplicated NS3 sequences that produce mature NS3 independent of the NS2 autoprotease activity showed increased replication and antigen expression. It seems likely that an alternative cellular cofactor controls NS2-3 cleavage and thus replication of APPV. The replication-optimized synthetic APPV genomes might be suitable live vaccine candidates, whose establishment and testing warrant further research.

## INTRODUCTION

Pestiviruses are the causative agents of many important animal diseases and form a distinct genus within the family *Flaviviridae*. A novel and very divergent pestivirus species of swine was discovered in 2015 and termed “atypical porcine pestivirus” (APPV) (1). Since then, APPV has been detected in the domestic pig population all over the world, including many countries of the Americas (2–8), Europe (9–19), and Asia (12, 20–31). RNA of the pathogen was found in preserved samples predating the prevalence of APPV in Europe to 1986 (16). The APPV was also detected in wild boar samples (17, 32–34), indicating an established virus reservoir in the wild. In 2017, the International Committee on Taxonomy of Viruses (ICTV) accepted APPV as a novel pestivirus species and named it *Pestivirus K* (35). APPV encodes the characteristic pestiviral proteins N^pro^ and E^rns^. However, in direct comparison to the other members of the genus, the nucleotide and amino acid sequences of APPV are highly divergent (36, 37). The polyprotein of APPV has only about 40% amino acid identity to the polyproteins of the classical pestiviruses species bovine viral diarrhea virus (BVDV-1 and -2; *Pestivirus A and B*), classical swine fever virus (CSFV; *Pestivirus C*), and border disease virus (BDV; *Pestivirus D*) (1).

Classical pestiviruses are able to cross the placental barriers and cause serious fetal disease. Fetal infections with noncytopathogenic (non-cp) BVDVs during the first trimester of gestation (day 0 to 125) may result in abortion, in mummification and stillbirth, or in malformation of the fetus, including cerebellar hypoplasia, skeletal malformation, hypotrichosis, and general growth retardation (38). However, surviving fetuses, which may also be clinically inconspicuous, become persistently infected (PI) and remain viremic throughout their lives. The establishment of persistent infections is limited to the maturation phase of lymphocytes, when T cells reactive against the body's own proteins are eliminated in the thymus (negative selection) to establish the so-called self-tolerance. If viral antigens are present in this sensitive phase of development, they are also recognized as “self.” Hence, PI animals show no adaptive immune response against the pestivirus, remain seronegative, and serve as a major virus reservoir (39). The detection and elimination of immunotolerant PI animals have been key to the control and eradication of BVDV in animal pest control programs in many European countries (40–42). APPV can establish diaplacental infections similar to the classical pestiviruses (43–46). A link between APPV *in utero* infections and the occurrence of congenital tremor (CT) type A-II in newborn piglets was established in epidemiological and clinical investigations (9, 10, 14, 47). The clinical picture of APPV CT A-II was experimentally reproduced using an APPV-containing piglet serum as an inoculum for the infection of gestating sows. Inoculation of gravid sows as well as experimental *in utero* infections of fetuses yielded neonatal piglets with detectable APPV loads in the serum and, in some cases, typical CT A-II symptoms (9, 47). The CT manifests as involuntary contractions of the skeletal muscles of variable intensity in these so-called “shaking piglets.” The disease pattern weakens as the piglets grow up, resulting in healthy-appearing adult pigs. In severe cases, the shaking piglets cannot suck milk from the teat and starve to death, resulting in economic losses in piglet production (48). Different studies suggested that APPV can establish persistent infections after these vertical transmission events, but despite strong evidence, definitive proof of APPV-immune tolerant piglets has not been established so far (14, 18).

The ability to evade the host's innate immune response is the prerequisite for viral persistence. Pestiviruses prevent stimulation of the innate immune response in several ways. They express two characteristic proteins that hinder the induction of the interferon system. The N-terminal autoprotease N^pro^ binds to interferon regulatory factor 3 (IRF3), prevents its binding to DNA, and promotes polyubiquitination and subsequent degradation (36, 49, 50). The secreted viral glycoprotein E^rns^ is an active enzyme that binds and degrades ssRNA with high efficiency. It was shown that E^rns^ also degrades double-stranded RNAs (dsRNAs) as well as highly structured single-stranded RNAs (ssRNAs) with lower efficiency, thus preventing the ssRNA- and dsRNA-induced type I interferon response in the infected cells (37, 51, 52). In addition, strict regulation of replication is necessary for the establishment of persistent infections to prevent cellular damage and not to kill the PI host.

The regulation of classical pestiviruses depends on a cellular cofactor of the NS2 autoprotease restricting the maturation of NS3 (53, 54). NS3 is a multifunctional viral protein with protease, helicase, and NTPase activities (55–57). While the mature NS3 is involved in genome replication, its uncleaved precursor, NS2-3, is essential for particle formation (58). An autoprotease activity located within the C-terminal domain of NS2 mediates the NS2-3 processing. The protease is activated by the cellular cofactor DNAJC14, also termed Jiv (J-domain protein interacting with viral protein) (53). After efficient NS2-3 processing at the onset of infection, the autoprotease activity of NS2 is restrained by the available cellular Jiv levels. This mechanism results in stably low viral replication, which characterizes the non-cp biotype of pestiviruses (54). Overexpression of Jiv in cultured cells deregulates the replication of non-cp pestiviruses and results in death of the infected cells.

A similar dysregulation is observed in the naturally occurring cytopathogenic (cp) biotype of pestiviruses. Some virus strains provide the Jiv cofactor themselves after integration of the cellular gene into the viral genome (59). Several other genetic alterations that cause increased release of mature NS3 have been identified, commonly leading to the emergence of cp strains. Prominent examples are autoactivating mutations within the NS2 protease, as well as deletions and duplications within the genome (45). Of particular interest are “defective interfering subgenomes” (DIs) with larger deletions, which rely on helper viruses for genome packaging (60–62). Pestiviral genomes with functional duplications of the NS3 gene usually produce NS2-3 and mature NS3 concurrently and form infectious particles independently. cp pestivirus genomes regularly emerge after natural mutation and selection in BVDV PI animals. The emergence of cp genomes leads to the death of the respective PI host due to a clinical syndrome called mucosal disease. However, the cp strains are unable to establish persistent infections and thus quickly disappear from the host population. Recent studies employed the CRISPR/Cas9 gene knockout technology to generate DNAJC14 gene knockout cell lines. These DNAJC14-deficient cells were resistant against the infection with non-cp strains of all tested pestivirus species, demonstrating that DNAJC14 is indeed an essential and universal cofactor of the pestiviral replication machinery (63).

Unlike the other pestiviruses, APPVs can be isolated and propagated in primary porcine cells and cell lines only with great difficulty. Despite these challenges, great progress has been made in the establishment of laboratory systems for the study of APPV in recent years. A first molecular clone of APPV allowing artificial infections and monoclonal antibodies (MAbs) against APPV NS3, which enables identification of the infected cells, has already been presented (64, 65). Furthermore, single strains of APPV have been adapted to continuous porcine cell lines. A recent study on such a cell culture-adapted APPV strain uncovered CD46 as a cellular receptor of APPV (66). The dependence on (co)receptors and cofactors might have been reduced or overcome by serial passages of the virus, because several amino acid changes occurred during cell culture adaptation. In addition to receptor binding and infection, replication of APPV in culture cells might also be adapted, as mutations were also present in the nonstructural proteins, especially in NS2 (66). Cultured cells might lack certain important host factors, or these factors might be present in insufficient amounts in comparison to viral target cells in the host animal, e.g., in the salivary glands or in fetal cells. In this study, we investigated the replication of APPV after transfection of synthetic RNA and tested the hypothesis that insufficient levels of DNAJC14, a conserved pestiviral host cell factor, could limit the propagation of APPV in cell culture.

## RESULTS

**FIG 1:**
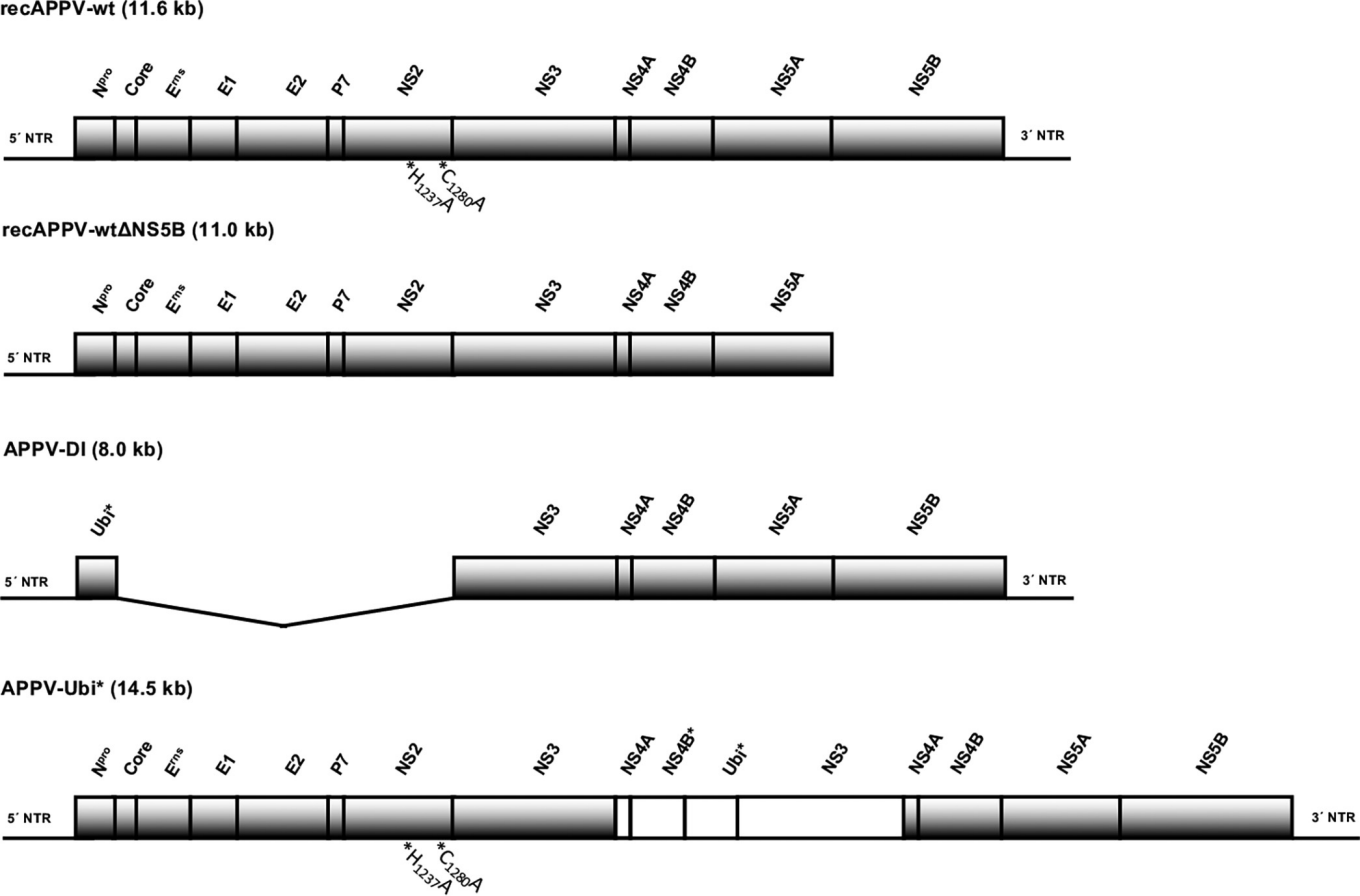
Scheme of the genome organization of the synthetic APPV genomes used in this study. NTR at the 5′ and 3′ ends of the genome and the mature processing products of the polyproteins are indicated. The labeled bars represent the N-terminal autoprotease (N^pro^), capsid protein (Core), glycoproteins (E^rns^, E1, and E2), and nonstructural proteins (p7 and NS2, -3, -4A, -4B, -5A, and -5B). A cDNA copy of an APPV field strain was cloned in a plasmid backbone and amplified by PCR, and infectious RNA was transcribed, generating recAPPV-wt. A nonreplicative subgenome was generated using a truncated PCR template. This negative control is lacking the NS5B gene sequence and the 3′ NTR and was named recAPPV-wtΔNS5B. To generate a replicative APPV-DI, we replaced the genes encoding N^pro^ to NS2 with a ubiquitin gene (Ubi*). Expression of mature NS3 in a complete genomic APPV was achieved by inserting Ubi* into NS5B gene sequences from the APPV-DI in frame with the truncated NS4 (NS4B*), resulting in APPV-Ubi*. The mutations H_1237_A and C_1280_A were introduced to inactivate the NS2 autoprotease.

The synthetic APPV genomes used in this study are depicted in Fig. 1.

### Monoclonal antibody N6 anti-APPV NS3.

**FIG 2:**
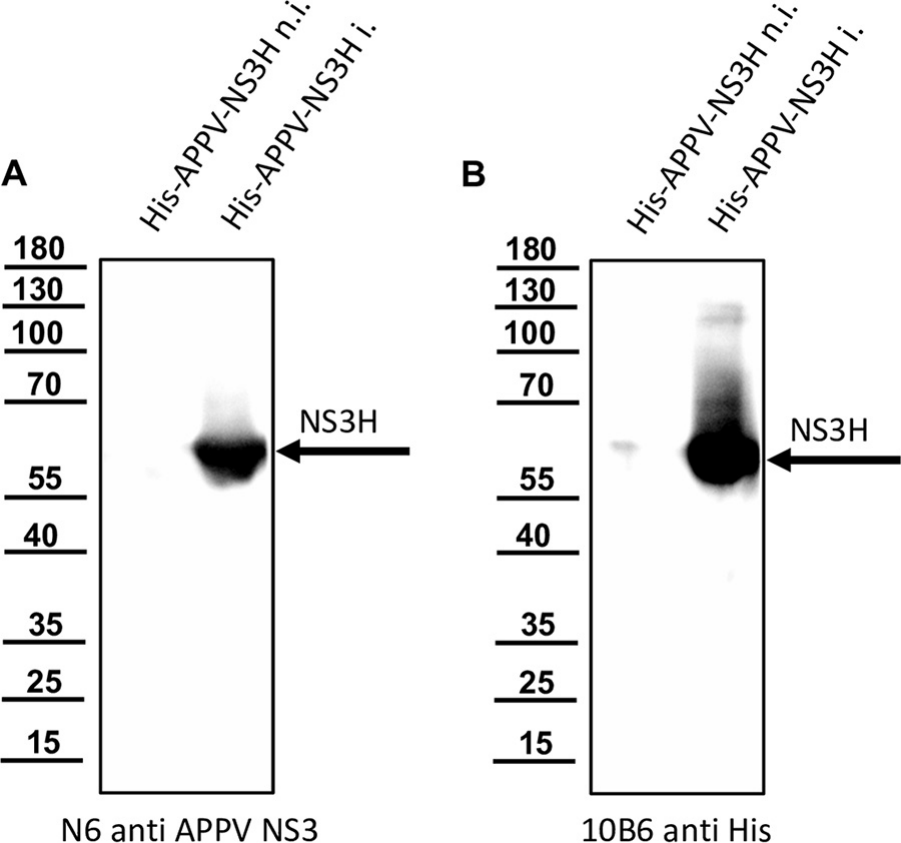
Reactivity of MAb N6 anti-APPV NS3 against recombinant NS3H expressed in E. coli. Total protein of noninduced (n.i.) and induced (i.) cultures of E. coli transfected with a pet11a vector encoding His-APPV-NS3H were resolved by SDS-PAGE and blotted onto a nitrocellulose membrane. The membranes were probed with MAb N6 anti-APPV NS3 (A) and MAb 10B6 anti-His (B). Both MAbs reacted with a single protein species with an apparent molecular weight of about 60 kDa (NS3H). The bands of a protein ladder are indicated on the left, in kilodaltons.

**FIG 3:**
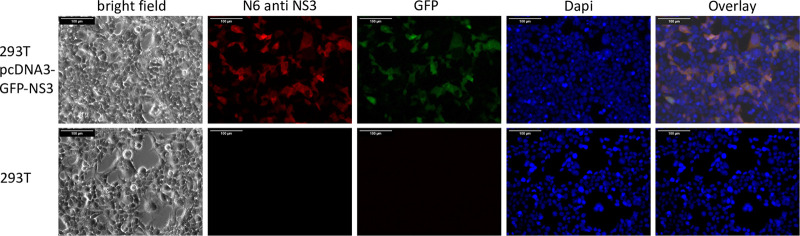
Reactivity of MAb N6 to APPV NS3 against recombinant GFP-NS3 expressed in 293T cells. A pcDNA3.1 plasmid encoding GFP-NS3 was transfected into 293T cells. Transfected and mock-transfected control cells were fixed, permeabilized, and probed with MAb N6 against APPV NS3. Binding of the MAb was visualized with a Cy3-labeled secondary antibody. A close correlation between Cy3 signals and GFP fluorescence was observed in the transfected cells, as demonstrated in the overlay picture with dominant yellow staining. No signals occurred in the mock-transfected cells. Cellular nuclei were localized with a DAPI stain. Bars, 100 μm.

We raised MAbs against APPV NS3 to unambiguously detect APPV infections of single cells in tissue culture. After immunization of mice with His-APPV-NS3H (14), cell fusion, and hypoxanthine-aminopterin-thymidine medium (HAT) selection, the supernatants of hybridoma cell clones were screened by an indirect enzyme-linked immunosorbent assay (ELISA). Hybridoma clone N6 was identified, secreting the ELISA-reactive anti-APPV NS3 MAb N6. MAb N6 was further characterized by immunoblotting using noninduced and induced cultures of Escherichia coli producing the His-tagged APPV NS3 helicase. MAb N6 detected a single protein band with an apparent molecular weight of 60 kDa in the induced culture (Fig. 2A). The anti-His-tag antibody 10B6 showed comparable reactivity in the control experiment (Fig. 2B). A green fluorescent protein (GFP)-tagged APPV NS3 (pcDNAGFP-NS3H) was used for immunofluorescence tests as described earlier (14). After transfection and expression of pcDNAGFP-NS3H in 293T cells, we tested MAb N6 using a Cy3-labeled goat-anti-mouse IgG for detection. We found a close correlation between GFP signals and the Cy3 immunostaining signals of MAb N6 in the transfected cells (Fig. 3). MAb N6 staining was more intense than GFP fluorescence, and therefore, it showed more antigen-positive cells. In nontransfected controls, neither green fluorescence nor red Cy3 staining was observed. To produce high-quality recombinant IgG, we determined the nucleic acid sequence of the complete mRNAs via reverse transcription-PCR (RT-PCR) and random amplification of cDNA ends-PCR (RACE-PCR) as previously described (67). The recombinant murine MAb N6 was produced from optimized genes using a pcDNA3.1 vector, T2A separation, and 293T cells. The IgG1 was purified from the cell culture supernatant and used for the experiments.

### Molecular clone of an APPV field strain.

**FIG 4:**
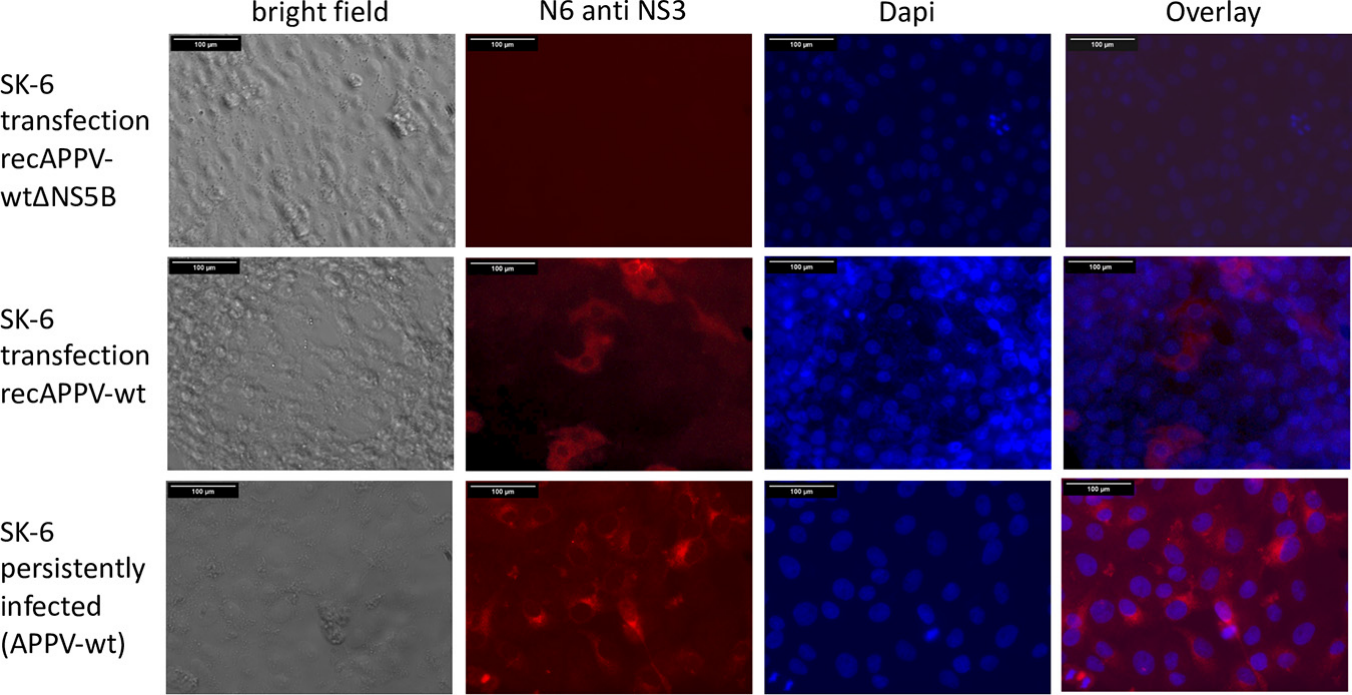
Molecular clone of an APPV field strain. SK-6 cells were transfected with synthetic RNA of recAPPV-wtΔNS5B (negative control) and recAPPV-wt by electroporation. SK-6 cells persistently infected with APPV field strain AUT-2016_C (APPV-wt) served as a positive control. After an incubation time of 48 h, the cells were fixed, permeabilized, and probed with MAb N6 anti-APPV NS3. A Cy3-labeled goat anti-mouse IgG was used for detection and a DAPI counterstain for nucleus localization. Dividing antigen-positive cells with characteristic cytoplasmic NS3 staining appeared in recAPPV-wt-transfected cells, and all cells were antigen positive in the persistently APPV-wt-infected cell line. Bars, 100 μm. Note the weaker and more focal staining in the persistently infected cells.

SK-6 cells persistently infected with the APPV field strain AUT-2016_C were selected using MAb N6. The cells were expanded and served as a source of viral RNA for our cloning attempts and as a positive control (APPV-wt). We amplified the complete genome of AUT-2016_C from total cellular RNA by RT-PCR. The genome was inserted in a pBR322 plasmid backbone containing a SP6 promoter in front of the first nucleotide of the APPV 5′ nontranslated region (NTR) and a MluI restriction enzyme cleavage site behind the genomes 3′ end. The clonal viral sequence was stable after passage of the plasmid as analyzed by high-throughput sequencing. Virus rescue was performed using PCR templates and SP6 mediated RNA transcription generating the infectious genomic RNA of the APPV wild-type (recAPPV-wt). A subgenomic RNA lacking the RNA-dependent RNA polymerase (RdRp) gene and the 3′ NTR was produced from a truncated cDNA and used as a negative control (recAPPV-wtΔNS5B). Transfection of the synthetic APPV RNA genomes in SK-6 cells using electroporation resulted in viral replication and antigen expression, as demonstrated by qRT-PCR and indirect immunofluorescence. Immunofluorescence signals of MAb N6 in recAPPV-wt-transfected cells (Fig. 4, recAPPV-wt) had low intensities but were slightly stronger than the signals in persistently APPV-wt-infected SK-6 cells (Fig. 4, APPV-wt). Transfection of the nonreplicative genome recAPPV-wtΔNS5B resulted in no antigen-positive cells (Fig. 4, recAPPV-wtΔNS5B).

Production of infectious viruses was analyzed by titration of sterilely filtered, cell-free culture supernatants in an endpoint dilution assay (EPDA) using naive SK-6 cells. As expected for an APPV wild-type genome, viral titers, measured as 50% tissue culture infectious doses (TCID_50_), were very low and never exceeded 10 TCID_50_/mL in both persistently APPV-wt-infected and recAPPV-wt-transfected cells. Infection experiments with the supernatant of persistently infected cells yielded 1 to 5 infected cells per mL, while the supernatant of recAPPV-wt transfected cells yielded 8 to 10 infected cells.

### APPV replicates efficiently in DNAJC14 knockout cells.

**FIG 5:**
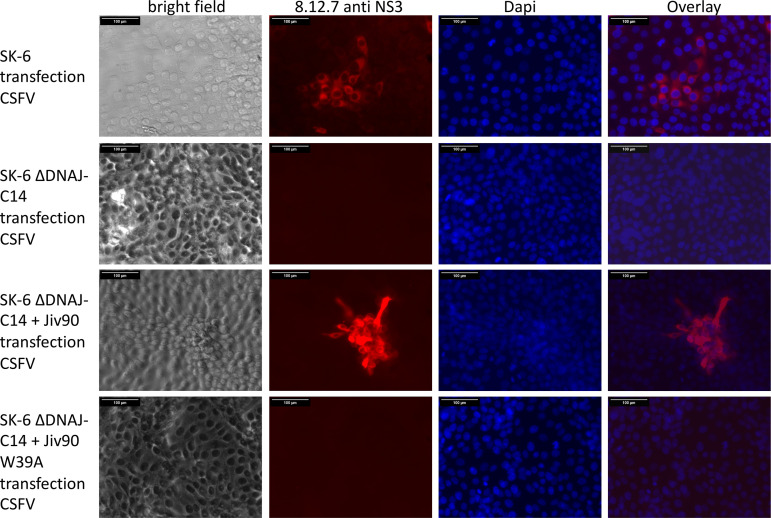
DNAJC14 dependency of CSFV. Transfection of synthetic RNA of CSFV (strain Alfort-Tuebingen) in SK-6 cells, SK-6 DNAJC14 knockout cells (SK-6 ΔDNAJC14), and SK-6 ΔDNAJC14 cells with knock-in of active Jiv90 (+ Jiv90) or inactive Jiv90 W_39_A (+ Jiv90 W_39_A). Transfection was done side by side with APPV RNAs as a control experiment (Fig. 6). At 48 h postelectroporation, the cells were fixed, permeabilized, and probed with MAb 8.12.7 against CSFV NS3. A Cy3-labeled goat anti-mouse IgG was used for detection and a DAPI counterstain for nucleus localization. Bars, 100 μm. CSFV antigen was detectable after transfection of RNA into SK-6 and SK-6 ΔDNAJC14 + Jiv90 cells. Note the strong antigen expression in SK-6 ΔDNAJC14 + Jiv90 and the induction of cytopathic effects. As described earlier, (63) the SK-6 ΔDNAJC14 and SK-6 ΔDNAJC14 + Jiv90 W_39_A were resistant to CSFV infection and showed no antigen expression.

**FIG 6:**
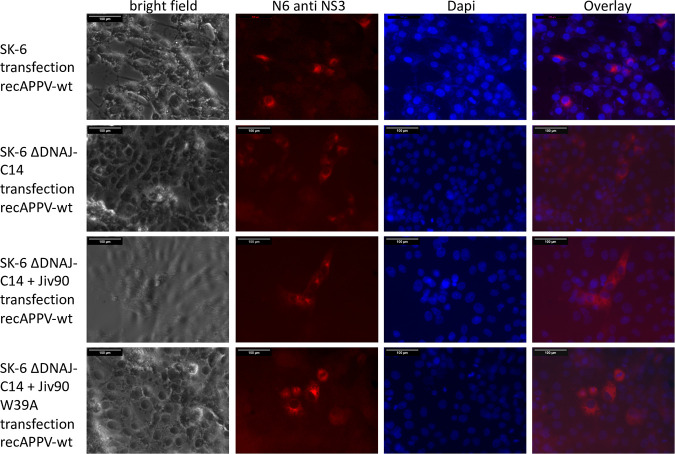
DNAJC14-independent replication of APPV. Transfection of synthetic RNA of recAPPV-wt in SK-6 cells, SK-6 ΔDNAJC14 cells, and SK-6 ΔDNAJC14 cells with knock-in of active Jiv90 or inactive Jiv90 W_39_A. At 48 h postelectroporation, the cells were fixed, permeabilized, and probed with MAb N6 against APPV NS3. A Cy3-labeled goat anti-mouse IgG was used for detection and a DAPI counterstain for nucleus localization. Bars, 100 μm. APPV antigen was detectable in all cell lines after transfection of recAPPV-wt RNA. Note that no CPE is visible in SK-6 ΔDNAJC14 + Jiv90 cells.

**FIG 7:**
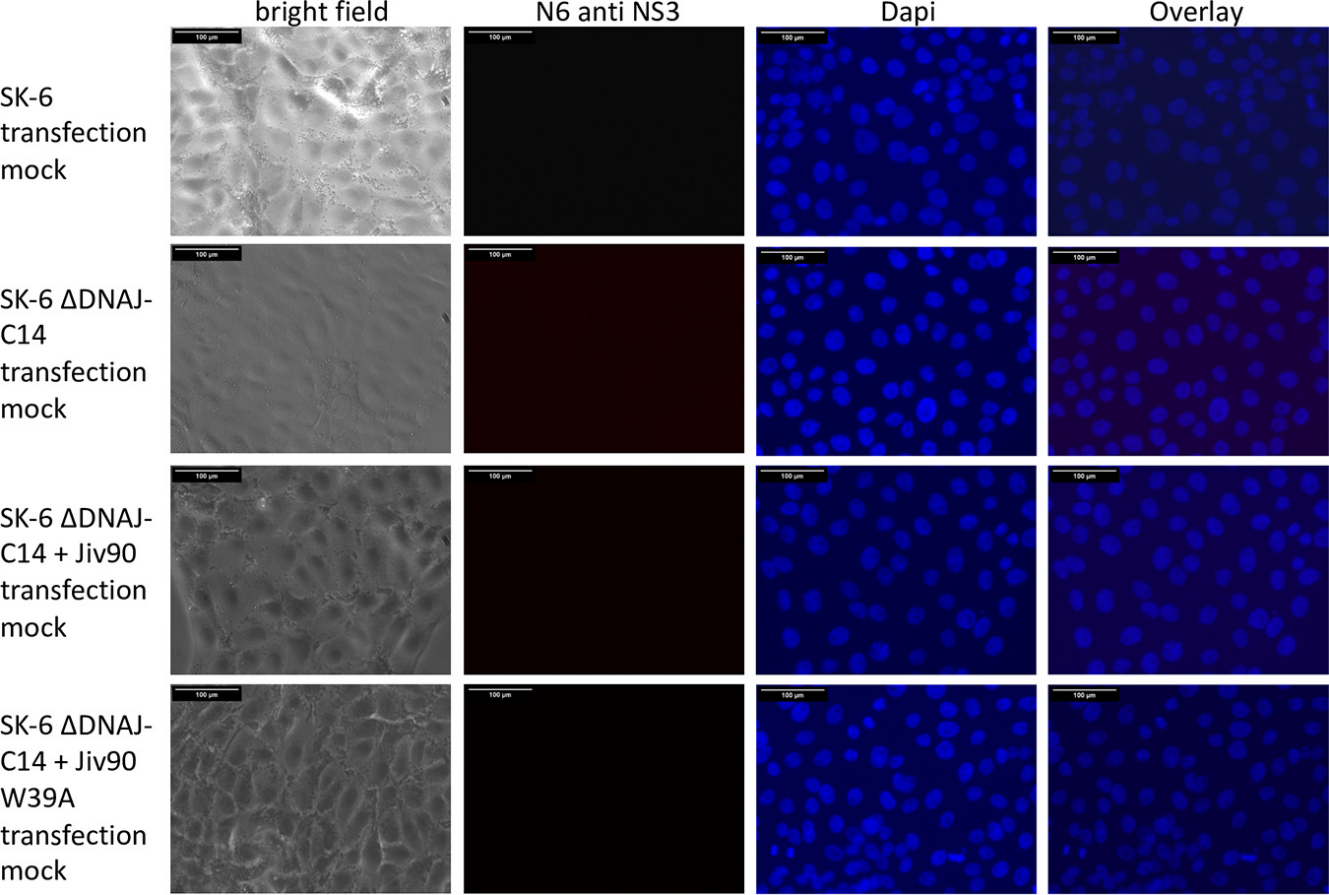
APPV NS3 is not detectable in mock-transfected cells (control experiment). SK-6 cells, SK-6 ΔDNAJC14 cells, and SK-6 ΔDNAJC14 cells with knock-in of active Jiv90 or inactive Jiv90 W_39_A were fixed, permeabilized, and probed with MAb N6 against APPV NS3. A Cy3-labeled goat anti-mouse IgG was used for detection and a DAPI counterstain for nucleus localization. Bars, 100 μm.

**FIG 8:**
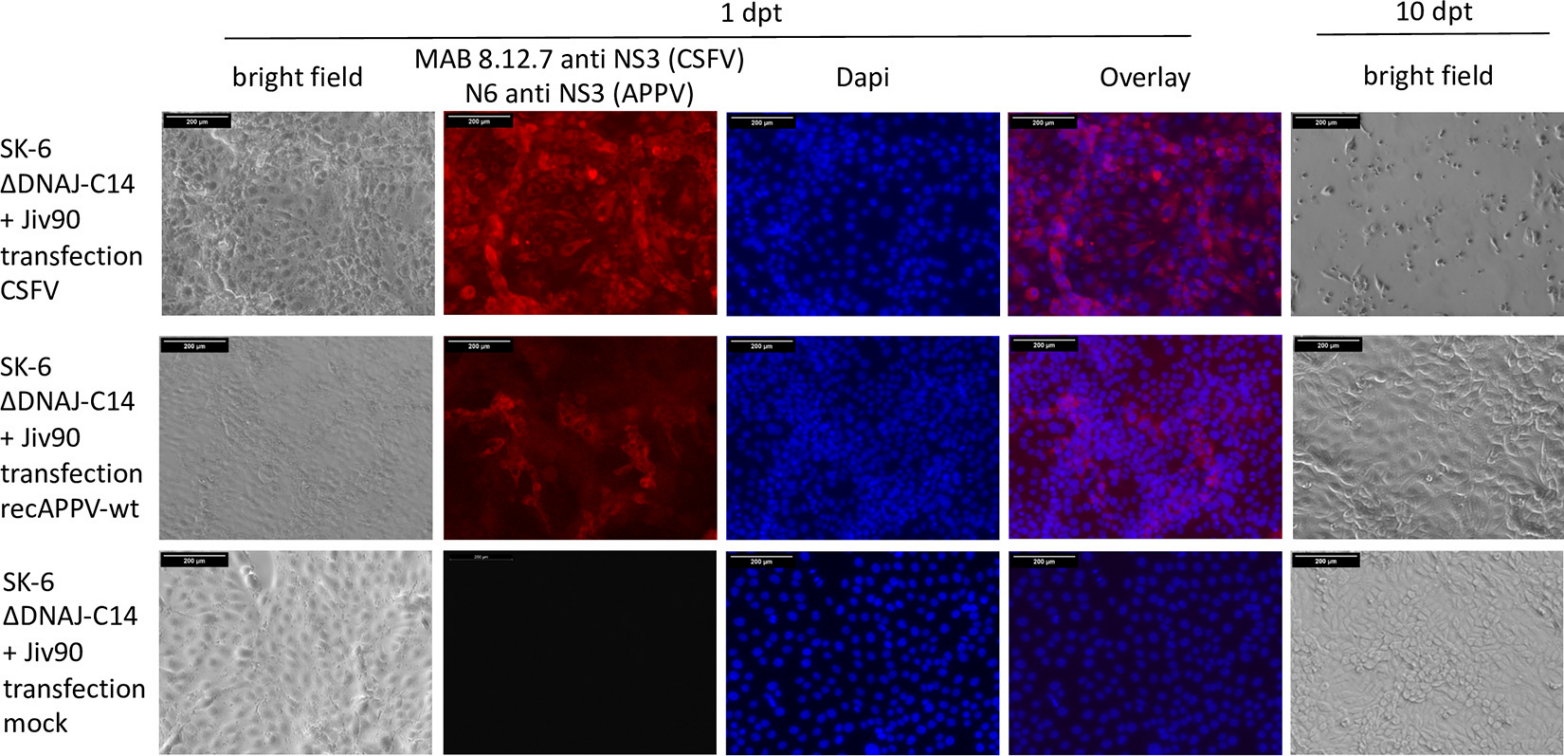
The recAPPV-wt genome replicates in SK-6 ΔDNAJC14 cells with knock-in of active Jiv90 without visible CPE. SK-6 ΔDNAJC14 cells with knock-in of active Jiv90 were transfected with CSFV (strain Alfort-Tuebingen) or recAPPV-wt or mock infected. An aliquot of the cells was stained at 24 h posttransfection using MAb 8.12.7 (CSFV), MAb N6 (APPV), or MAb 8.12.7 and N6 (Mock) to verify the success of the transfection. A Cy3-labeled goat anti-mouse IgG was used for detection and a DAPI counterstain for nucleus localization. Bars, 100 μm. Another aliquot of the cells was passaged and documented in a bright-field image at day 10 posttransfection. Note that the cellular monolayer is completely destroyed in CSFV-infected SK-6 ΔDNAJ-C14 + Jiv90 cells, while no obvious changes are visible in recAPPV-wt transfections in comparison with the mock infection control.

The transfection experiment clearly showed that electroporation of synthetic RNA initiates the replication cycle of APPV and that there is no principal resistance of the cells. However, it must be considered that large amounts of RNA are introduced into the cells in this experiment, so that the process differs considerably from natural virus infection. We wanted to investigate whether an increased dependence on the cellular cofactor DNAJC14 hindered APPV infection/propagation in cell culture. Our hypothesis was that DNAJC14 levels present in cultured cells were insufficient to initiate replication of a single RNA molecule after virus infection but sufficient to initiate replication after artificial transfection. Therefore, we used the synthetic RNA of recAPPV-wt to transfect the DNAJC14-deficient SK-6 knockout cell lines, as well as the DNAJC14 knockout/knock-in counterparts overexpressing highly active or inactive DNAJC14 variants. After transfection of recAPPV-wt RNA, we monitored viral replication by indirect immunofluorescence assays. Recombinant RNA of CSFV (strain Alfort-Tuebingen) was used in a control experiment. The CSFV replicated efficiently in the parental SK-6 cell line but showed no replication in the DNAJC14 knockout cells and in inactive Jiv90 W_29_A variant knock-in cells (Fig. 5), as described previously (63). Surprisingly, the transfection of recAPPV-wt RNA led to viral replication, antigen expression, and immunofluorescence signals of APPV in DNAJC14 knockout and knock-in cell lines. Comparable immunofluorescence signals of MAb N6 were apparent in parental SK-6 cells, DNAJC14 knockout SK-6 cells, and Jiv90 cells as well as Jiv90 W_29_A knock-in cells after transfection (Fig. 6). APPV NS3 antigen was not detectable in the immunostaining using MAb N6 of the mock-transfected cells (Fig. 7). This indicates that the APPV replication is independent of the availability of DNAJC14, at least in the cell culture model. As expected, the CSFV replicated with a cp phenotype in the SK-6 knock-in cells that overexpressed the highly active DNAJC14 variant Jiv90, as was clearly visible at 10 days after transfection by the complete destruction of all host cells (Fig. 8). In contrast, the transfection of recAPPV-wt RNA in SK-6 knock-in cells that overexpressed the highly active DNAJC14 variant Jiv90 led to viral replication in the absence of visible cellular damage even after 10 days posttransfection.

### APPV replication levels are increased in response to expression of mature NS3.

**FIG 9:**
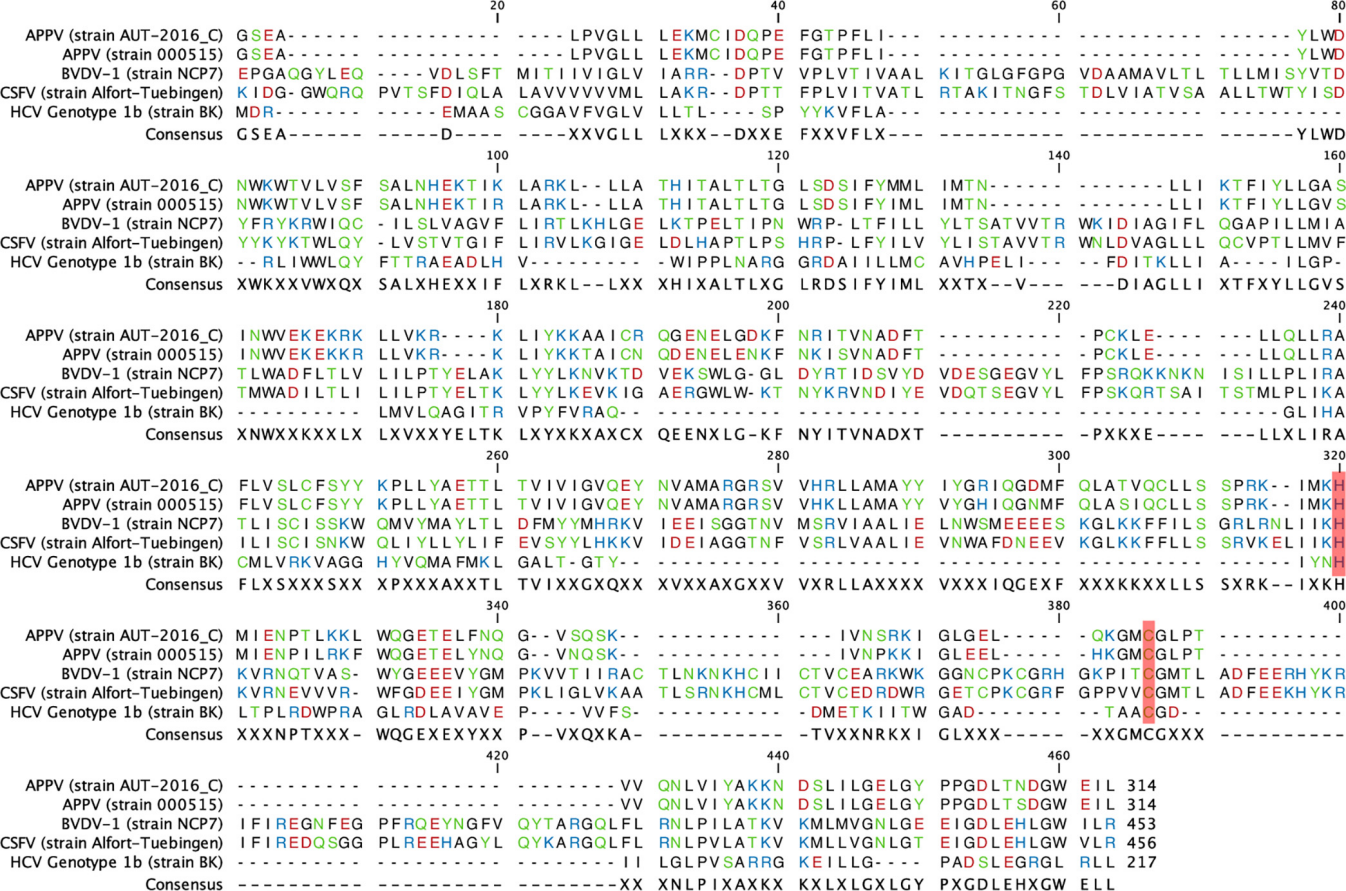
Alignment of pestiviral NS2 sequences. The NS2 sequences of APPVs (strain AUT-2016_C and 000515), BVDV-1 (strain NCP7), CSFV (strain Alfort/Tuebingen), and HCV (strain BC) were aligned using the program CLC Workbench with default settings. The proposed active-site residues of the NS2 autoprotease C_1280_ and H_1237_ are highlighted in red. Amino acids are labeled with polarity colors to display homologies between the sequences. A consensus sequence is displayed using the “majority” setting and an X as symbol for ambiguous residues. Note the poor homologies and similarities between the sequences, which are almost exclusively restricted to the C-terminal protease domain.

**FIG 10:**
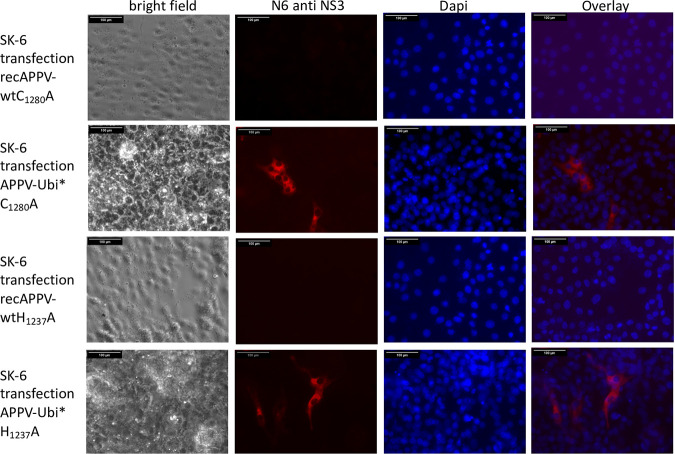
The single-amino-acid changes C_1280_A and H_1237_A completely abolish the replication ability of recAPPV-wt, while APPV-Ubi* is not affected. SK-6 cells were transfected with synthetic RNA of recAPPV-wtC_1280_A and APPV-Ubi*C_1280_A (top rows) as well as recAPPV-wtH_1237_A and APPV-Ubi*H_1237_A (bottom rows) by electroporation. The cells were fixed, permeabilized, and probed with MAb N6 against APPV NS3 after an incubation of 48 h. A Cy3-labeled goat anti-mouse IgG was used for detection and a DAPI counterstain for nucleus localization. Bars, 100 μm. No antigen-positive cells appeared in the recAPPV-wtC_1280_A and recAPPV-wtH_1237_A images, while APPV-Ubi*C_1280_A and APPV-Ubi*H_1237_A were not affected by the mutation. The experiment indicates a functional relevance of these residues for NS2-3 cleavage and the importance of NS3 release for APPV.

Since DNAJC14 seems to be not essential for the replication of APPV, the question of whether NS2-3 cleavage is important for APPV arises. First, we wanted to know whether inactivation of the NS2 autoprotease by replacement of the active site residues affects the replication of APPV. We inserted the mutations C_1280_A and H_1237_A into the putative protease domain of NS2, generating recAPPV-wtC_1280_A and recAPPV-wtH_1237_A (Fig. 9). After transfection of both NS2 mutants, no antigen-positive cells were detected by MAb N6 staining (Fig. 10). Consequently, it can be assumed that the identity of the two amino acids in the active site of the NS2 protease is essential for APPV replication.

**FIG 11:**
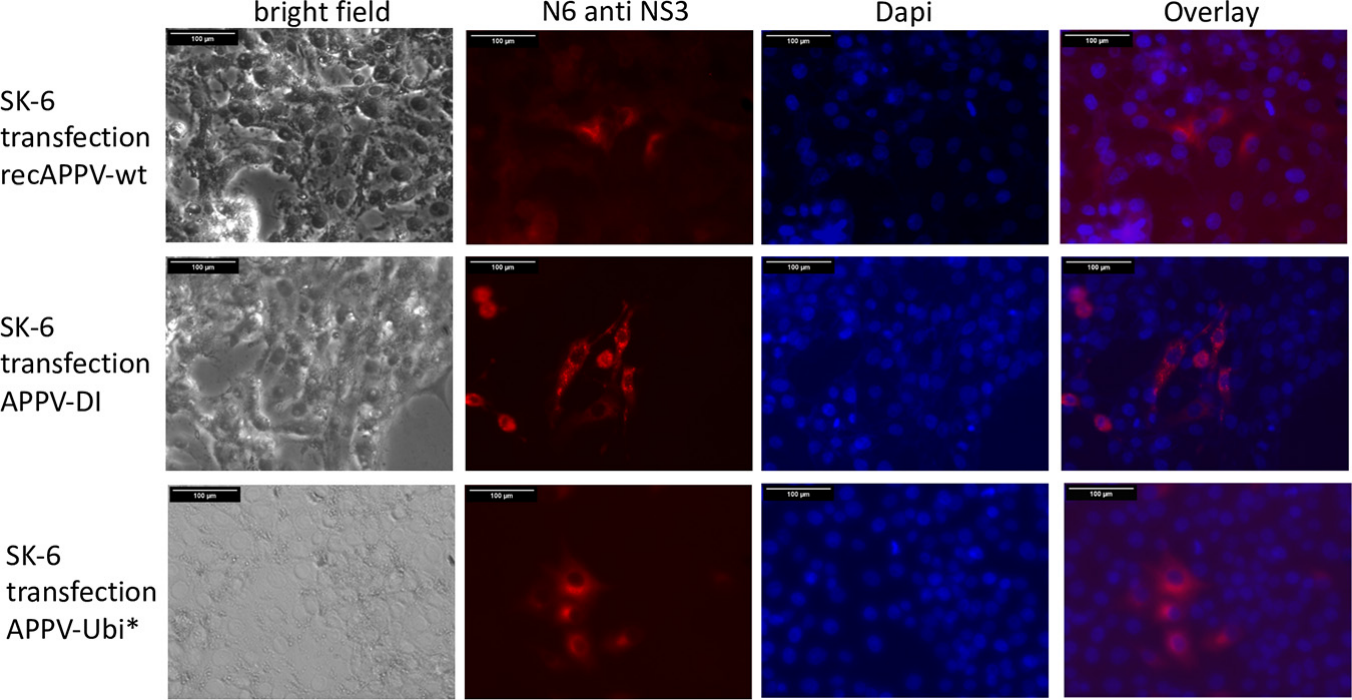
APPV genomes expressing mature NS3 independently of the NS2 autoprotease. SK-6 cells were transfected with synthetic RNA of recAPPV-wt, APPV-DI, and APPV-Ubi* by electroporation. The cells were fixed, permeabilized, and probed with MAb N6 anti-APPV NS3 after an incubation of 48 h. A Cy3-labeled goat anti-mouse IgG was used for detection and a DAPI counterstain for nucleus localization. Bars, 100 μm. Very intense staining of dividing cells was observed for subgenome and the genome with duplicated NS3 sequences, while a weaker staining is visible in the wild-type genome. Note that no CPE is visible in antigen-positive cells transfected with APPV genomes expressing mature NS3 independently of NS2 autoprotease.

**FIG 12:**
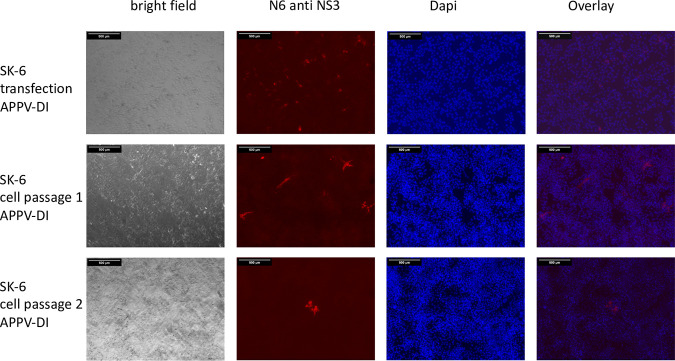
APPV-DI is not cytopathogenic. SK-6 cells were transfected with synthetic RNA of APPV-DI by electroporation. Aliquots of the transfected cells were fixed at 48 h posttransfection as well as after passages 1 and 2 of the cells. After permeabilization, the cells were probed with MAb N6 against APPV NS3 and a Cy3-labeled goat anti-mouse IgG. A DAPI counterstain was used for nucleus localization. Bars, 500 μm. A smaller magnification had to be chosen to show the formation of foci and to show their decreasing number. Note that cells transfected with the DI continue to divide and form multicellular foci.

**FIG 13:**
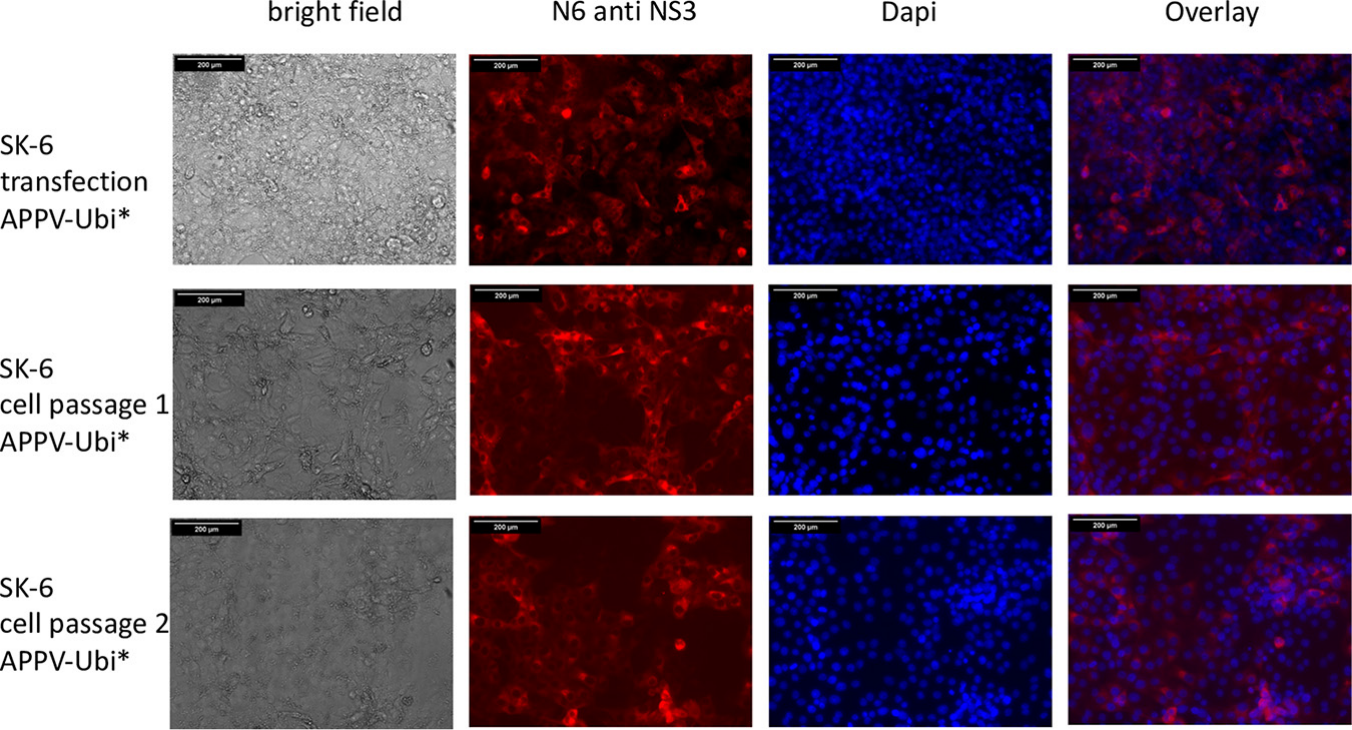
APPV-Ubi* is not cytopathogenic. SK-6 cells were transfected with synthetic RNA of APPV-Ubi* by electroporation. Aliquots of the transfected cells were fixed at 48 h posttransfection as well as after passages 1 and 2 of the cells. After permeabilization, the cells were probed with MAb N6 against APPV NS3 and a Cy3-labeled goat anti-mouse IgG. A DAPI counterstain was used for nucleus localization. Bars, 100 μm. Note that the cells transfected with APPV-Ubi* still appear healthy in the cell passages and that their number remains constant.

We further generated a mutant genome with duplicated viral sequences that produce mature NS3 independent of the NS2 autoprotease and also inserted the NS2-inactivating mutations C_1280_A and H_1237_A as a control, which showed strong antigen signals. To directly compare the antigen expression of NS2-3- and NS3-expressing genomes, we electroporated synthetic RNAs of recAPPV-wt, an APPV-DI, and APPV-Ubi* (Fig. 11) side by side. While weak fluorescence signals were observed after transfection of the recombinant APPV-wt RNA and MAb N6 staining, stronger signals were present in the cells transfected with the subgenomic APPV-DI, APPV-Ubi*. Surprisingly, no cytopathogenic effects (CPE) appeared in APPV-DI and APPV-Ubi* transfected cells, which is usually observed in DIs and free NS3 expressing strains of pestiviruses. Cells with ongoing replication of (sub)genomes of APPV, which express mature NS3, could readily be passaged (Fig. 12). The only striking feature in the case of subgenomic APPV-DI that could indicate a noxious effect was that the number of antigen-positive cells slowly decreased in control staining with MAb N6. However, remaining foci of antigen-positive cells indicated ongoing cellular proliferation. In contrast, in the case of APPV-Ubi*, a constant number of antigen-positive cells was observed, which instead increased from passage to passage (Fig. 13). This could be correlated with the (minimal) release of infectious particles into the supernatant by APPV-Ubi*, which is absent in the case of the subgenomic DI. However, the production of infectious virus progeny in cell cultures increased only minimally in the APPV-Ubi* within 3 passages, progressing from 0 TCID_50_/mL (P1) to values not exceeding 20 TCID_50_/mL (P3). It can be assumed that genetic adaptations to the imbalance between NS2-3 and free NS3 led to the increase in titers, since the NS2 mutations in APPV-Ubi*C_1280_A and APPV-UbiH_1237_A already released infectious particles at passage 1, also producing no more than 20 TCID_50_/mL.

**FIG 14:**
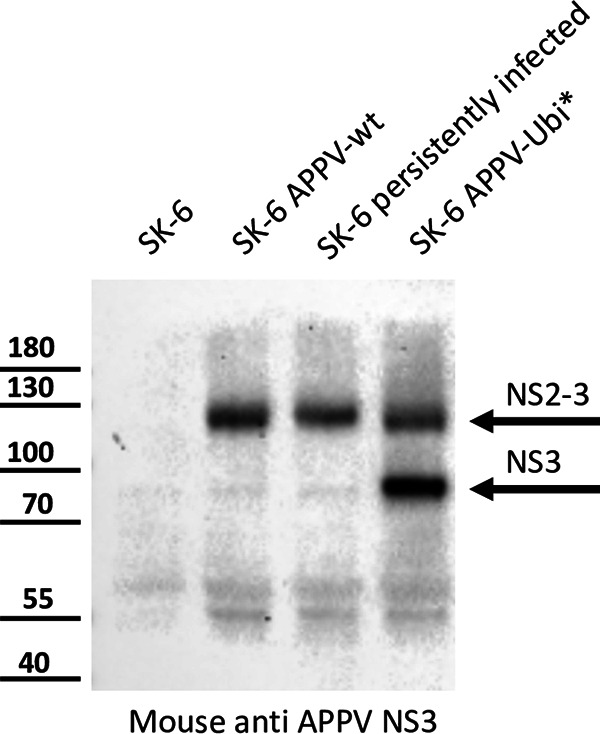
Analysis of NS2-3 processing of APPV-wt and APPV-Ubi* using immunoblotting. Mock-infected SK-6 cells, cells transfected with RNA of recAPPV-wt, persistently infected cells, and APPV-Ubi*-transfected cells were harvested at 48 h posttransfection. Cellular proteins were extracted by detergent lysis buffer, concentrated using ultrafiltration, mixed with loading buffer, and resolved by SDS-PAGE. The proteins were blotted on a nitrocellulose membrane and probed with a polyvalent mouse antiserum against APPV NS3. Nonspecific background reactions can be identified in the mock-infected SK-6 cells at about 80 kDa. An 80-kDa band is also seen in the cells transfected with the recAPPV-wt and in the persistently infected cells, but the intensity is only slightly stronger than in the mock infection control. A strong band appears at approximately 80 kDa in the APPV-Ubi*-transfected cells, which can be assigned to mature NS3 with confidence. Another band appears in the APPV-Ubi*- and APPV-wt-transfected cells as well as in the persistently infected cells at approximately 120 kDa. This band can be assigned to the NS2-3 precursor protein and could additionally contain uncleaved NS2-3-4A precursors. A protein band at about 55 kDa might represent a helicase fragment of the NS3, which has been described for CSFV and many other flaviviruses. On the left, the protein size standard (in kilodaltons) is included for direct comparison.

A Western blot assay using a polyvalent mouse antiserum demonstrated the expression of NS2-3 (apparent molecular weight of 120 kDa, calculated molecular weight 112.5 kDa) and mature NS3 (apparent molecular weight of 80 kDa, calculated molecular weight of 77 kDa) in APPV-Ubi* (Fig. 14). It also showed the NS2-3 of recAPPV-wt and of APPV-wt (apparent molecular weight of 120 kDa). However, we could not detect the mature NS3 of wild-type APPVs with confidence. A very weak Western blot band could represent NS3, but a background band was detected at the same time in the lysate of naive SK-6 cells. However, the detectable amounts of NS2-3 and mature NS3 in the APPV-wt-infected cells were weak in comparison to the APPV-Ubi* amounts, in accordance with the weaker signals seen in the immunofluorescence assays.

**FIG 15:**
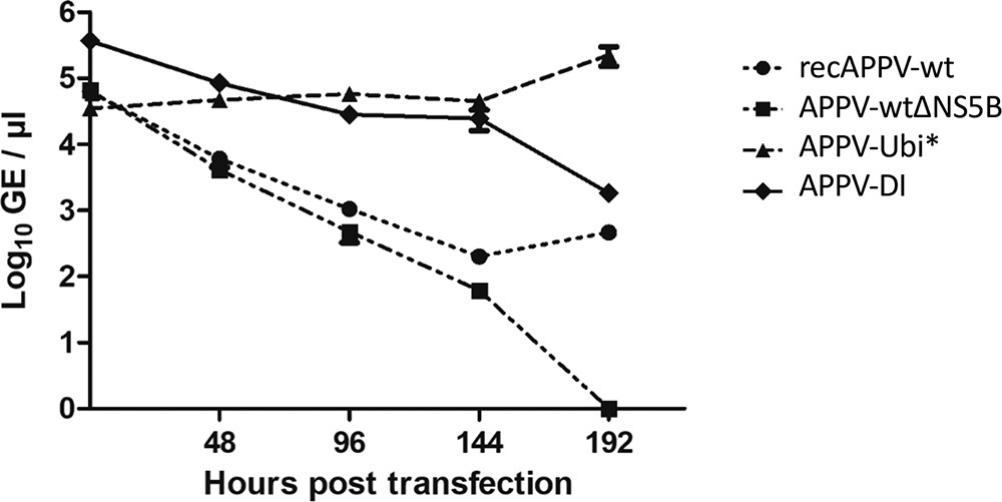
Replication of synthetic RNAs of recombinant APPV genomes. SK-6 cells were transfected with synthetic RNA of recAPPV-wt, recAPPV-wtΔNS5B (negative control), APPV-DI, or APPV-Ubi*. One half of each transfection reaction was seeded into a separate well of a 6-well plate. After the cells had settled, one of these wells was washed and harvested, providing the 0-h value. The other well was further incubated and trypsinized every 48 h. The cells were split in a 1:2 ratio. One half of the cells was reseeded, while the other half was harvested for analysis. Extracellular synthetic RNAs and DNAs were removed from the harvested cells by Benzonase treatment. RNA was extracted and measured in an APPV-specific RT-qPCR assay. Genome equivalent values were calculated based on a standard curve of purified synthetic RNA. Detectable amounts of nucleic acids from the nonreplicative subgenome APPV-wtΔNS5B were present until 144 h posttransfection. In direct comparison of the curves, active replication of APPV-DI and APPV-Ubi* became apparent at 48 h, while the APPV-wt needed 96 h to exceed the background of synthetic RNA. Elevated RNA levels were documented for APPV-DI and APPV-Ubi*.

Since viral protein expression levels indicated a difference in the RNA replication of the APPV-wt and the mutant genome APPV-Ubi* encoding mature NS3, we decided to measure the viral RNA replication of these genomes within the transfected cells. In a direct comparison between the nonreplicative subgenome APPV-wtΔNS5B, in which the levels of intracellular synthetic RNA constantly declined and fell below the detection limit at 192 h, replication of the APPV-wt genome became evident at 144 h posttransfection (Fig. 15). In a further comparison, we observed approximately 100-fold-higher intracellular RNA levels at 144 h and 1,000-fold higher RNA levels at 192 h posttransfection in APPV-Ubi* genome-transfected cells. A similar curve was obtained from APPV-DI, where intracellular RNA levels remained relatively constant after transfection but then began to decrease at 144 h posttransfection. Similarly, a decrease in the number of antigen-positive cells was observed in the control immunofluorescence staining as presented above.

**FIG 16:**
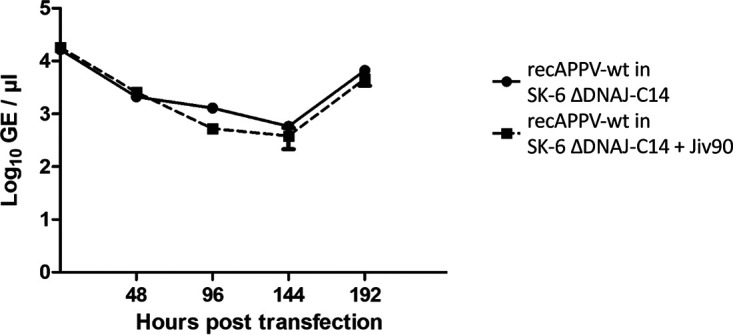
Replication of synthetic RNAs of recAPPV-wt in SK-6 ΔDNAJC14 and SK-6 ΔDNAJC14 with knock-in of active Jiv90. Cells were transfected with synthetic RNA of recAPPV-wt by electroporation. One half of each transfection reaction was seeded into a separate well of a 6-well plate. After the cells had settled, one of these wells was washed and harvested, providing the 0-h value. The other well was further incubated and trypsinized every 48 h. The cells were split in a 1:2 ratio. One half of the cells was reseeded, while the other half was harvested for analysis. In a direct comparison of the curves, no significant differences in APPV genome replication between SK-6 ΔDNAJC14 and SK-6 ΔDNAJC14 with knock-in of active Jiv90 is visible.

We further compared the intracellular RNA levels of the recAPPV-wt in DNAJC14 knockout SK-6 cells and the Jiv90 knock-in counterparts (Fig. 16). Using aliquots of a single RNA synthesis reaction, we obtained very similar curves showing a clear increase in RNA levels at 192 h posttransfection. The curves also correspond nicely to the replication curve obtained before from the recAPPV-wt replicating in the genetically unmodified SK-6 cell line (Fig. 15).

### Secreted RNAs of recAPPVs lack infectivity.

**FIG 17:**
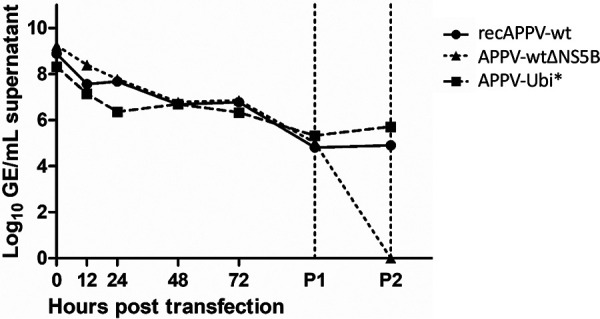
Measurement of viral RNA in the cell culture supernatant. Cells were transfected with synthetic RNA of recAPPV-wt, the nonreplicative subgenome APPV-wtΔNS5B, and APPV-Ubi* by electroporation. Samples from the cell culture supernatant were taken at the indicated time points and after passages 1 and 2 of the transfected cells. Accessible RNAs and DNAs were removed from the harvested supernatant by Benzonase treatment. In a direct comparison of the curves, no significant differences in APPV genome equivalent numbers are visible between recAPPV-wt, the nonreplicative subgenome APPV-wtΔNS5B, and APPV-Ubi* before passage 2. Note the significant amount of secreted viral RNA in passage 2.

Using our reverse genetics system, we found no evidence of restriction of APPV genome replication in the cultured Sk-6 cells. However, we could barely detect infectivity secreted by the cells transfected with recAPPV-wt in our cell culture model. We now wanted to determine whether viral morphogenesis or viral infection was impeded in the cultured cells. Therefore, we quantified the amount of secreted RNAs after transfection in the cell culture supernatant (Fig. 17). Because of the large amounts of synthetic input RNA, we decided to digest free nucleic acids in the supernatant by Benzonase treatment before RNA extraction. However, we could not detect the secretion of viral RNAs over the synthetic RNA background without passaging the transfected cells twice. Even after passaging once using trypsinization and fresh medium, no difference was detectable between the nonreplicative subgenome (APPV-wtΔNS5B) and recAPPV-wt or APPV-Ubi*. Electroporation appeared to produce Benzonase-resistant RNA-protein complexes or slowly released, cell-associated RNA-containing membrane vesicles. Not until cell passage 2 was the background of the APPV-wtΔNS5B nonreplicative subgenome below the detection limit, whereas both recAPPV-wt and APPV-Ubi* produced approximately 10^6^ secreted genome equivalents per mL, which were protected against Benzonase digestion. The release of viral RNA was measured previously in the supernatant of APPV-infected cultures and in the sera of infected animals, which also showed hardly any infectivity (14). Our data may be taken as evidence of functional viral morphogenesis and virus shedding, because low doses of infectivity were found, while considerable amounts of RNA were released into the supernatant. Thus, the lower infectivity of APPV particles more likely results from problems with attachment, receptor-mediated endocytosis, membrane fusion, or uncoating. Future studies will address these issues.

## DISCUSSION

APPV can be unambiguously assigned to the genus *Pestivirus* based on many different criteria. In addition to the typical genome organization with the conserved NS3 protease cleavage sites in the nonstructural-protein region, it encodes the characteristic proteins N^pro^ and E^rns^, which are unique to pestiviruses. Host preference and disease pattern also perfectly match, because APPV is able to cause diaplacental infections in a cloven-hooved animal species, leading to neurological disorders (9, 10, 14, 47). However, there are also a number of features that distinguish this atypical porcine pestivirus from the other known species. Apart from a truncated receptor-binding envelope protein (E2), a very large overall sequence divergence must be noted. It is also unusual that propagation of the pathogen in cultured cells seems to be possible only after adaptation, since the other known pestiviruses grow without an adaptation in primary and many different permanent cell lines of their respective host species.

In this study, we established and used a complete laboratory system for APPV to investigate the replication of this unusual pestivirus in cell culture. The detection of viral protein expression in the host cells is pivotal for any virological study, to allow quantification of infectious loads and to monitor the infection process at the single-cell level. Polyvalent porcine sera against APPV obtained from recovered pigs have been used in multiple studies (66), but in our hands, these led to rather weak specific signals and high background levels. Such sera could be purified by affinity chromatography using recombinant viral proteins, as demonstrated earlier by our group (14). However, isolation of specifically binding polyclonal antibodies from antisera by affinity chromatography is laborious, requires extensive characterization of the individual batches, and yields limited quantities of these reagents. Hence, we decided to generate NS3-specific mouse antisera and MAbs to overcome these limitations and reliably detect APPV infections of our cultured cells. MAb N6 anti-APPV NS3 was extensively characterized, including by ELISA, immunofluorescence assays, Western blotting, sequence determination and recombinant production. Although similar reagents have been presented before by others (65), we can now offer MAb N6 as a reagent with broad applicability.

Because APPVs do not grow in cell culture without an adaptation, the transfection of synthetic RNA was chosen as an elegant way to study replication of a nonadapted APPV wild-type genome. The nucleotide sequence of APPV strain AUT-2016_C (GenBank no. KX778724) obtained from an infected piglet was presented earlier (14). Only single nucleotide changes became apparent when comparing this published consensus sequence with the sequence of the clonal cDNA copy generated in this study (GenBank no. OK564403). The virus used for molecular cloning was isolated from a different animal, and errors that may exist in the previously published consensus sequence cannot be excluded or evaluated on a hypothetical basis. Hence, we now consider the sequence of the molecular clone as authoritative for strain AUT-2016_C because it has a proven functionality. Electroporation of the RNA into susceptible host cells resulted in antigen expression detectable in immunofluorescence, genome replication measurable by qRT-PCR, and production of (few) infectious progeny virus. Future studies will investigate adaptation of the recombinant virus strain to cell culture using recloning of genome fragments.

We found that APPV can replicate independently of the host cell factor DNAJC14. The replication of classical pestiviruses in their host cells is limited by the availability of this cellular cofactor, which controls the release of NS3 and thus the maturation of active proteins (53, 54). A stable, low viral replication level is the characteristic of the non-cp biotype of pestiviruses. These regulatory mechanisms can be overcome by different mutations resulting in cofactor-independent NS3 release. Such genomes replicate unrestrained and form the cp biotype of pestiviruses, which causes host cell death after infection (54). Some naturally occurring cp pestivirus strains provide the DNAJC14 cofactor themselves after an integration of the cellular mRNA into their viral genome (59). Overexpression of DNAJC14 in cultured cells deregulates the replication of all “classical” non-cp pestiviruses in a similar way by activating the NS2 autoprotease, resulting in large amounts of mature NS3, enhanced RNA replication and cytopathogenicity. Recent studies employed the CRISPR/Cas9 gene knockout technology to generate bovine and porcine DNAJC14 gene knockout cell lines. These DNAJC14-lacking cells were resistant against infection with non-cp strains of all tested pestivirus species, demonstrating that DNAJC14 is an essential and universal cofactor of the pestiviral replication machinery (63). cp strains of pestiviruses, on the other hand, were able to replicate in the DNAJC14-lacking cell lines because they generate mature NS3 independently of this host factor.

To investigate whether DNAJC14-dependent processing of the NS2-3 precursor is also important for APPV replication, we used the established DNAJC14 knockout SK-6 cells in APPV RNA transfection experiments. After electroporation of recAPPV-wt RNA into the DNAJC14 knockout cell line, we detected regular replication and protein expression. The expression of APPV NS3 was visualized by indirect immunofluorescence, and RNA replication was demonstrated by RT-PCR. Importantly, no replication of CSFV was seen in control experiments with the DNAJC14 knockout cells. The replication in the absence of DNAJC14 indicates a fundamental difference in the regulation of NS2-3 cleavage of APPV in comparison to all other pestiviruses. The independence of APPV from DNAJC14 as a host cell factor was further substantiated by the use of the control knock-in cells, which overexpressed a highly active 90-amino-acid fragment of DNAJC14, called Jiv90 (53). The overexpressed Jiv90 activates the NS2 autoprotease and accelerates the replication of non-cp pestiviruses to cytopathic levels, as demonstrated by visible CPE in a CSFV control experiment. In contrast, replication of recAPPV-wt RNA was not enhanced by Jiv90 overexpression, and no CPE appeared in the transfected cells.

To increase our knowledge about the regulation of APPV replication, we introduced several mutations. First, we prepared a nonreplicative subgenomic viral RNA lacking the RdRp-encoding gene and the 3′ NTR as a negative control. This control showed that antigen expression of a nonreplicative synthetic APPV RNA remained below the detection limit of our immunofluorescence assays. Therefore, in immunofluorescence-positive cells, active genome replication can be inferred. We further used this control RNA in our qRT-PCR assays and found that active replication of recAPPV-wt genomes after transfection of synthetic RNA was rather difficult to measure directly. After transfection, synthetic nonreplicative RNAs and/or contaminating cDNA remnants from the transcription reaction produced a background in the quantitative RT-PCR (RT-qPCR) assays that dropped below the detection limit only after passage and several days of culture. The high background due to synthetic input RNA occurred in both intracellular RNA and secreted RNA measurements and could not be eliminated by Benzonase treatment of the cell culture supernatant. We suspect exocytosis of RNA-containing vesicles that might be formed as part of repair processes after electroporation.

We further tested the importance of NS2-3 cleavage for APPV replication and individually mutated the two proposed active-site residues of the NS2 autoprotease to alanine. As transfection of recAPPV-wtC_1280_A and recAPPV-wtH_1237_A mutants did not lead to antigen-positive cells, we can conclude that the identity of the residues and the activity of the NS2 autoprotease are pivotal for APPV replication. In addition, we found strong evidence that the two amino acids are located in the active site of the autoprotease and catalyze the proteolytic reaction, as shown in the sequence alignment (Fig. 9) and previously inferred from homology to classical pestiviruses by others (1).

In addition to an integration of the DNAJC14 gene, several other genetic alterations that cause increased release of mature NS3 lead to the emergence of the cp biotype of classical pestiviruses. Of importance are activating mutations within the NS2 autoprotease, as well as deletions and duplications within the genome. Some replication-competent defective interfering subgenomes exhibit the loss of genes located at the 5′ end of the genome, rely on helper viruses for genome packaging, and cause CPE, because the NS2 gene is deleted. Pestiviral genomes with functional duplications of the NS3 gene provide both NS2-3 and mature NS3 in parallel and can independently form infectious particles. We investigated whether APPV is also capable of this biotype switch typical for the other pestiviruses. We generated an APPV subgenome using the cp BVDV DI subgenome DI-9 as a template. We fused the 5′ NTR of APPV directly to a ubiquitin-coding cassette, which was followed by the nonstructural genes NS3-NS4A-NS4B-NS5A-NS5B and the 3′ NTR. We further constructed APPV-Ubi*, a replication- and packaging-competent genome with a duplicated NS3 region modeled on the cpBVDV strain CP Rit/4350, in which the processing of NS3 is uncoupled from the autoprotease activity of NS2. As with other pestiviruses, the availability of mature NS3 increased the replication levels of APPV RNAs in case of APPV-DI and APPV-Ubi*. Despite 1,000-fold-increased amounts of viral RNA and significant increases in viral protein levels in APPV-DI- and APPV-Ubi*-transfected cells, no obvious effects on the viability of the host cells were observed. The transfection of APPV-DI and APPV-Ubi* resulted in the generation of persistently infected cells, demonstrating a non-cp biotype of these genomes. Since replication and protein expression of classical cp pestiviruses induce apoptosis, it can only be speculated whether APPV lacks apoptosis-inducing factors or whether some nonstructural proteins of APPV may have antiapoptotic effects on the host cell. Future studies could address this question through protein expression together with apoptosis-inducing compounds.

Interestingly, the NS2-3 cleavage and replication of APPV are independent of the canonical flaviviral cofactor DNAJC14, which broadly modulates flavivirus replication. Whereas in pestiviruses, only NS2-3 cleavage depends on the interaction with DNAJC14, reduced and elevated levels of DNAJC14 result in replication inhibition in viruses of the genus *Flavivirus* mediated by membrane modulations during replication complex formation (68). In the case of APPV, recruitment of a different cellular cofactor to regulate NS2-3 cleavage and replication seems likely, because we could show that NS2-3 cleavage is important for APPV replication and our experiments gave evidence for high-level regulation of NS2-3 cleavage. While the NS2-3 is detectable in cell lysates of APPV-wt-infected cells, the amounts of mature NS3 are very low and can be reliably detected by Western blotting only in the APPV-Ubi* mutant. Such a strict regulation of the polyprotein processing has been described in non-cp BVDVs, where mature NS3 is not detectable in Western blots. The consistently low replication rates of APPV after establishment of infection strongly hint at a negative feedback loop or a coupling of viral replication to the constitutive homeostasis of the cell. The identification of a now suspected alternative cellular cofactor of the APPV NS2 autoprotease is very complex, and this needs to be pursued in future studies.

Unfortunately, one key question about APPV could not be addressed in this study. By using a molecular clone, we were able to bypass the lack of infectivity of APPV-containing samples and create a cell culture model, but we were unable to determine the exact cause of the lack of infectivity. While our studies only suggest that APPV virions are inhibited in cell culture models at early steps of the infection cycle, other groups have already made further progress and have been able to characterize cell culture-adapted genomes (66). Because APPV strains are genetically highly variable, it is difficult to transfer the described mutations in the viral structural proteins to the genome presented here. We therefore plan to adapt our molecular clone on the SK-6 cells and confirm the data in an independent system using a different strain. Of particular interest is the question of the cellular receptor, since CD46 should in principle be present on all cultured cells.

Although much knowledge is already available on the unusual atypical porcine pestiviruses, many important questions about the biology of APPV remain also to be investigated. In particular, the question of the induction of immunotolerant PI animals should be followed. This can be answered with reliability only by controlled infection studies using gestating sows and long-term follow-up of the diaplacentally infected offspring using clonal virus genomes from cell culture or defined produced virus stocks originating from reverse genetic systems. Furthermore, it should be investigated in detail whether and to what extent the common endemics in farms cause economic damage in pig production. In this context, the possibility that APPV strains with different degrees of virulence exist cannot be excluded, so identification of genetic factors and measurement of virulence in animal experiments seem necessary. In addition to the direct negative effects on the animals, indirect effects, such as suppression of the immune system of affected animals, should also be considered and investigated in immunological studies. Last but not least, an effective prophylaxis and surveillance program should be established to prevent introduction of the virus into APPV-free farms, which has been shown to cause problematic losses.

The findings of this study and our newly developed tools could help to clarify many of these questions. On the one hand, recombinant antigens, the MAb, and the expression-optimized viral genomes could be used to establish sensitive serological tests. On the other hand, the recombinant APPV strains might serve as a platform to analyze the pathogenicity and virulence of APPV field strains in the natural host. In addition, the APPV-Ubi* strain presented here could represent a first promising model for an effective live vaccine. Modeled after the long-used vaccine strain BVDV CP Rit, the APPV-Ubi* genome is most likely attenuated and will cause a stronger immune response than unmodified wild-type genomes due to its superior protein expression. Of course, this would have to be clarified in controlled animal experiments, in which not only acute infections of adult animals but also diaplacental infections of fetuses would be evaluated.

## MATERIALS AND METHODS

### Cells, serological reagents, viruses, and RNA transfection.

SK-6 cells, a swine kidney cell line (69), and 293T cells, a human cell line that expresses a mutant version of the SV40 large T antigen (ATTC, CRL-3216), were grown in Dulbecco’s modified Eagle’s medium (DMEM) supplemented with 10% heat-inactivated fetal calf serum (FCS). Cells were maintained at 37°C and 5% CO_2_. SK-6 cell lines with DNAJC14 gene knockout (DNAJC14-KO), DNAJC14-KO cells overexpressing Jiv90 (DNAJC14-KO+Jiv90), and DNAJC14-KO overexpressing the inactive variant Jiv90W_39_A (DNAJC14-KO+Jiv90W_39_A) were described earlier (63) and were maintained as described in this publication (63). The murine anti-APPV NS3 MAb N6 is presented in this study. A polyclonal mouse anti-APPV NS3 serum was generated (Davids Biotechnologie, Regensburg, Germany) and used for Western blot detection of APPV NS3 as indicated. MAb 8.12.7 against pestivirus NS3 is an established antibody and was used for CSFV detection (70). The molecular clone of CSFV, plasmid p447 (strain Alfort-Tuebingen), was used as a control (71). The APPV strain AUT-2016_C was chosen for the molecular cloning of an APPV field strain, because a nearly complete genomic sequence and suitable control samples were available (14). Polyprotein amino acid numbers throughout this study refer to the sequence of this strain (KX778724). Viruses were rescued from synthetic infectious RNA as previously described (72). Briefly, SK-6 cells were transfected with 0.1 to 1 μg of synthetic RNA by electroporation using a single pulse of 950 μF and 0.18 kV in a 0.2-cm cuvette (Gene Pulser; Bio-Rad, Feldkirchen, Germany).

### Generation and characterization of serological reagents against APPV NS3.

Murine MAbs and a polyclonal antiserum against APPV NS3 were generated using a recombinant NS3 antigen (His-APPV-NS3H), which was presented earlier (14). Briefly, the helicase domain of NS3 (amino acids [aa] 1513 to 2006) was expressed using the pet11a vector system in the E. coli strain Rosetta 2 (Novagen, Madison, WI, USA). The His-tagged NS3 helicase domain with a calculated molecular weight of 57.8 kDa was purified by ion metal affinity chromatography (IMAC) using Ni^2+^-Sepharose (HisTrap HP; GE Healthcare, Chicago, IL, USA). Protein preparations were further purified using size exclusion chromatography (Superdex 200 10/300; GE Healthcare) and served as antigen sources for immunizations and ELISA screenings.

Immunizations were performed in BALB/c mice (Janvier Labs, Le Genest-Saint-Isle, France) applying a mild adjuvant (Gerbu MM; GERBU, Heidelberg, Germany) according to the manufacturer's specifications. All animal use protocols employed in this study were approved by the institutional ethics and animal welfare committee and the Austrian authority according to paragraphs 26 and following of the “Animal Experiments Act” from 2012 (permit number BMWF-68.205/0074-WF/V/3b/2017).

After immunizations, hybridoma cell clones were generated using standard methods (73). Freshly prepared spleen cells were fused with sp2/0-AG14 myelomas with the help of polyethylene glycol (PEG), seeded, and selected by aminopterin, hypoxanthine, and thymidine medium. Secreted MAbs from crude cell culture supernatants were characterized by ELISA, Western blotting, and immunofluorescence assays, leading to the identification of hybridoma cell line N6, secreting MAb N6. The isotype of MAb N6 was determined from cell culture supernatant as IgG1 heavy chain and kappa light chain using the Iso-Gold rapid mouse monoclonal isotyping kit (Dianova). The mRNAs of IgG1 heavy chain and kappa light chain were amplified with subtype specific oligonucleotide sets as described previously (67). After 5′-end and 3′-end determination by RACE-PCR (FirstChoice; Fisher Scientific, Schwerte, Germany), the mature mRNAs were cloned in a T vector and sequenced (Microsynth, Balgach, Switzerland). Optimized synthetic genes were designed, separated by a teschovirus 2A peptide sequence, and commercially cloned into a single pcDNA3.1 expression vector (BioCat, Heidelberg, Germany). The plasmid was transfected in 293T cells with polyethylenimine (PEI) using standard methods. MAb N6 was harvested from serum-free cell culture supernatant and purified using protein G affinity chromatography (HiTrap Protein G HP; Cytiva, Marlborough, MA, USA). The recombinant purified N6 was used for the detection of APPV NS3 unless explicitly stated otherwise.

### Indirect immunofluorescence assay and Western blotting.

Indirect immunofluorescence assays were performed as described earlier (74). Cells were fixed with 4% paraformaldehyde for 20 min at 4°C, permeabilized with 0.5% (vol/vol) Triton X-100 (Merck, Darmstadt, Germany) in phosphate-buffered saline (PBS), stained using the respective MAbs, and visualized using goat anti-mouse IgG conjugated with Cy3 (Dianova, Hamburg, Germany) or goat anti-mouse IgG conjugated with fluorescein isothiocyanate (FITC; Dianova) in PBS with 0.05% (vol/vol) Tween 20 (PBS-T; Invitrogen, Karlsruhe, Germany). Cellular nuclei were counterstained with DAPI (4′,6-diamidino-2-phenylindole; Thermo Fisher Scientific, Waltham, MA, USA) at a concentration of 1 μg/mL for 5 min at room temperature. Antigen detection was evaluated on a very sensitive fluorescence microscope (IX70; Olympus, Tokyo, Japan), and photomicrographs were taken using a monochromatic camera (DFC3000G; Leica, Wetzlar, Germany). All images in which information comparing replication rates is provided were taken in the same experiment with identical microscope settings.

For Western blotting, cells were directly harvested in SDS-PAGE loading buffer and heated at 95°C for 5 min. Only in the case of Western blot detection of APPV NS3 from infected cells, regular protein extraction (1% NP-40, 0.5% Triton X-100, 0.1% SDS in PBS) and sample concentration (Sartorius Vivaspin 20; molecular weight cutoff [MWCO], 30 kDa; polyethersulfone) were performed beforehand. Proteins were separated in 7.5% (wt/vol) polyacrylamide Tricine gels and transferred onto nitrocellulose membranes (Pall, Pensacola, FL, USA). The membranes were blocked with 5% (wt/vol) skim milk (Carl Roth, Karlsruhe, Germany) in PBS-T. MAbs and sera were used as indicated and detected by peroxidase-labeled goat anti-mouse IgG (Dianova). ECL-Prime substrate was applied as chemiluminescence reagent (GE Healthcare), and photon emission was recorded with an imaging system (ChemiDoc; Bio-Rad, Hercules, CA, USA).

### Persistently infected cell cultures.

Despite initial infection of single cultured cells, field isolates of APPV usually show no spread in tissue cultures. Therefore, SK-6 cells persistently infected with isolate AUT-2016_C were generated using a modified virus isolation technique. A serum sample of an AUT-2016_C-infected piglet which died before colostrum uptake was used to infect SK-6 cells in suspension. These cells were incubated for 1 h, diluted in DMEM to a concentration of 10 cells/mL and seeded on 96-well plates using 100 μL per well. After clonal proliferation, the cells were harvested by trypsinization and split to another corresponding plate. The mirror plates were fixed with formalin after 2 days and subjected to immunostaining using the APPV NS3 specific antibody N6. Single APPV-infected cell lines emerged on each plate. The corresponding wells containing APPV-infected SK-6 cell clones were harvested and reseeded for proliferation. Persistently infected cell clones were used for the experiments and stored in liquid nitrogen.

### APPV cDNA clones.

A culture of the persistently infected SK-6 cells was harvested and RNA was prepared by a QiaCube using the RNeasy kit (Qiagen, Hilden, Germany). Viral loads were measured as genome equivalents (GEs) using RT-qPCR. Based on the sequence information of APPV-AUT-2016_C (14) and other available APPV sequences from databases (in particular, strain APPV-NL1; KX929062), we designed a full-genome RT-PCR protocol. The complete APPV genome was amplified using the OneTaq one-step RT-PCR kit (New England Biolabs [NEB], Ipswich, MA, USA) with the oligonucleotides APPV-5′_forw and APPV-3′_rev (Table 1). The 11,564-bp RT-PCR product was inserted into a pBR322-derived vector (73). The vector containing the SP6 promoter was amplified by PCR. The PCR product provided homologous sequence patches for the molecular cloning of the viral cDNA. Q5 polymerase (NEB) was used for vector amplification together with the oligonucleotides APPV-pBRGA_forw and APPV-pBRGA_rev. The PCR and RT-PCR products were purified (Monarch DNA gel extraction kit; NEB) and recombined using a DNA assembly reaction (NEBuilder; NEB) to generate recAPPV-wt_pBR (Fig. 1). The plasmid DNA was purified and sequenced after transformation and clonal replication in bacteria (E. coli strain HB101). Sequencing was repeated after 10 rounds of plasmid DNA purification and retransformation, proving the stability of the construct. The sequence was compared with the reference sequence of the APPV strain AUT-2016_C (KX778724).

**TABLE 1:** Oligonucleotides used in this study

Oligonucleotide	Sequence
APPV-5′_forw	5′-GCATAATGCTTTGATTGGCTGCATTATG-3′
APPV-3′_rev	5′-GGGCCCCCTTGCTTCATCTAGATCAG-3′
APPV-pBRGA_forw	5′-ATGAAGCAAGGGGGCCCACGCGTTACCTCACTAACGTTC-3′
APPV-pBRGA_rev	5′-CAATCAAAGCATTATGCTATAGTGTCACCTAAATCGC-3′
APPV-5′-NTR_rev	5′-CATGTTGATAAACAACGGATTTTATAC-3′
APPV-NS3_forw	5′-GGCCCCGGCAGAATACCAAAGATCAC-3′
APPV-5′-NTR-Ubi*_forw	5′-TGTTTATCAACATGGAAGGAGATGACTATGC-3′
APPV-NS3-Ubi*_rev	5′-GGTATTCTGCCGGGGCCCCCACCACGAAGTCTCAACAC-3′
SP6-BACGA_rev	5′-GCCAATCAAAGCATTATGCTATAGTGTCACCTAAATCGTTA-3′
APPV-3′-BACGA_forw	5′-GATGAAGCAAGGGGGCCCTAAATAGCTTGGCGTAATCATG-3′
APPV-NS4B*_rev	5′-TCTCTTAGCATAGTCATCTCCTTC-3′
APPV-NS4B*-Ubi*_forw	5′-ATGACTATGCTAAGAGAGAGAATGGCAAAATCAGTCGC-3′
CmRGA_forw	5′-GTACTGTTGTAATTCATTAAGCATTCTGCCGAC-3′
CmRGA_rev	5′-GTCGGCAGAATGCTTAATGAATTACAACAGTAC-3′
APPV-C1280A_forw	5′-CAAAAGGGCATGGCCGGCCTCCCAACTGTAGTACAAAATTTG-3′
APPV-C1280A_rev	5′- TACAGTTGGGAGGCCGGCCATGCCCTTTTGCAATTCCCCCAG-3′
APPV-H1237A_forw	5′-ATTATGAAAGCCATGATAGAGAATCCAACTCTCAAGAAG-3′
APPV-H1237A_rev	5′-TGGATTCTCTATCATGGCTTTCATAATTTTTCTCGGGCTTGAC-3′
APPV-qRT_forw^*a*^	5′-GGGCAGACGTCACYGAGTAGTACA-3′
APPV-qRT_rev^*a*^	5′-TCCGCCGGCACTCTATCA-3′
APPV-probe^*a*^	FAM-5′-TGTAGGGTCTACTGAGGCT-3′-MGB
SP6-5′_forw	5′-CGATTTAGGTGACACTATAG-3′
APPV-NS5A-3′_rev	5′-CAATTTAAGGTGGCTGGCGTATCTTTC-3′

a Described by Kaufmann et al. (16).

A subgenomic defective interfering genome (DI) was constructed in analogy to the genomic organization of the BVDV defective interfering genome DI9 (60). The N^pro^ gene, the structural protein genes (C, E^rns^, E1, and E2), and the nonstructural protein genes p7 and NS2 were replaced in the plasmid by a ubiquitin gene cassette derived from the BVDV strain CP-Rit (75). The vector was amplified by Q5 PCR using the oligonucleotides APPV-5′-NTR_rev and APPV-NS3_forw, while the ubiquitin gene insert was amplified by One-Taq polymerase and the oligonucleotides APPV-5′-NTR-Ubi*_forw and APPV-NS3-Ubi*_rev. The DNAs were recombined, and the resulting plasmid was named APPV-DI_pBR.

Since genomic duplications turned out not to be stable in the context of a normal plasmid (for example, pBR322), a different strategy was chosen to generate duplication mutants. Hence, the APPV clone with a duplication of the NS3-to-NS4B cassette was constructed in a bacterial artificial chromosome (BAC) backbone as described for CSFV (74). The BAC backbone (pBeloBac11; NEB) was amplified using the oligonucleotides SP6-BACGA_rev and APPV-3′-BACGA_forw and Q5 polymerase (NEB). The APPV genome was amplified from recAPPV-wt_pBR as described above and recombined with the BAC backbone using DNA assembly, resulting in the plasmid recAPPV-wt_BAC. Plasmid recAPPV-wt_BAC was used for the insertion of the duplicated genes, which were inserted in close analogy to the genome of BVDV strain CP-Rit. The BAC backbone, including the APPV 5′ NTR, N^pro^, the structural protein genes, and the nonstructural protein genes of p7, NS2, NS3, and NS4A and of a fragment of NS4B (NS4B*) was amplified using Q5 polymerase and oligonucleotides APPV-NS4B*_rev together with APPV-3′-BACGA_forw. A subgenomic cassette including the Ubi*-Rit gene, all nonstructural protein genes, and the 3′ NTR was amplified from APPV-DI_pBR using the oligonucleotides APPV-NS4B*-Ubi*_forw and APPV-3′_rev. Recombination of the two PCRs using NEBuilder resulted in plasmid APPV-Ubi*_BAC.

For the generation of single site mutations within the APPV-wt genome, we used the BAC clone recAPPV-wt_BAC and employed one assembly site for homologous recombination in the chloramphenicol acetyltransferase gene (CmRGA_forw and CmRGA_rev) within the vector and complementary overlapping oligonucleotides encoding the respective mutations. Two different amino acid changes were introduced in the recAPPV-wt_BAC and APPV-Ubi*_BAC to inactivate the NS2 autoprotease. The plasmids recAPPV-wtC_1280_A_BAC and APPV-Ubi*C_1280_A_BAC were created with the oligonucleotides APPV-C1280A_forw and APPV-C1280A_rev, and the plasmids recAPPV-wtH_1237_A_BAC and APPV-Ubi*H_1237_A_BAC were created with the oligonucleotides APPV-H1237A_forw and APPV-H1237A_rev. All mutations were verified using Sanger sequencing and a commercial provider (Eurofins Genomics, Ebersberg, Germany). An overview of the synthetic APPV genomes used in this study is given in Fig. 1.

### qRT-PCR.

An established diagnostic RT-qPCR protocol (16) was adapted for genome equivalent quantification in cell culture using APPV-qRT_forw, APPV-qRT_rev, and APPV-probe. GEs were calculated by 7500 System SDS software (Applied Biosystems, Foster City, USA) based on a standard curve of purified synthetic genomic RNA of recAPPV-wt (as described in “Synthesis of recombinant viral RNA”). The viral RNA genome replication of different recombinant APPV strains was quantified after transfection using qRT-PCR. Therefore, the transfected cells were trypsinized every 48 h after electroporation and split in a 1:2 ratio. One half of the cells was reseeded on a 6-well dish, while the other half was resuspended in buffer and digested for 1 h with 1 μL Benzonase at 37°C (>250 IU; Sigma-Aldrich, Taufkirchen, Germany) to remove extracellular synthetic RNA and DNA. Total cellular RNA was prepared using the RNeasy minikit (Qiagen). To evaluate the release of viral RNA into the cell culture supernatant, supernatant samples were taken at regular time intervals after RNA transfection and extracted using the QIAamp viral RNA minikit (Qiagen).

### Synthesis of recombinant viral RNA.

Synthetic viral RNA was prepared using SP6 polymerase and PCR products of the respective plasmids or BACs as the template. Genome-length PCRs were performed using the pBR based plasmid clones (recAPPV-wt_pBR and mutants) as well as the BAC based clones (recAPPV-wt_BAC and mutants) using oligonucleotides SP6-5′_forw and APPV-3′_rev together with Q5 polymerase (NEB). We included a subgenomic PCR as a template generating a nonreplicative negative-control RNA using oligonucleotides SP6-5′_forw and APPV-NS5A-3′_rev. This subgenomic negative-control RNA was lacking the complete NS5B polymerase and the 3′ NTR (recAPPV-wtΔNS5B) (Fig. 1). PCR products were purified using phenol-chloroform extraction. The linear DNA fragments with a 5′-end SP6 promoter sequence were transcribed into genomic RNA using SP6 polymerase (NEB) as recommended by the manufacturer. Reaction products transcribed at a 50-μL scale were digested with DNase and purified using the RNeasy minikit (Qiagen). The RNA was eluted from the column in RNase-free water, quantified, and adjusted to a final concentration of 0.1 μg per μL.

### TCID_50_ assay.

The TCID_50_ of viral supernatants was determined in three replicates by an EPDA using a monolayer of SK-6 cells and 24-well plates. After 4 days postinfection, the cells were fixed and stained as described above. Solely single infected cells became visible using 100 μL undiluted culture supernatant. The virus titers were calculated using the Spearman-Karber algorithm.

### Data availability.

The sequence of the anti-APPV NS3 MAb construct with proven functionality and the sequence of the clonal cDNA copy generated in this study were deposited in the GenBank database (accession no. OK564404 and OK564403). Upon request, we can also provide the expression plasmid or hybridoma cells as a control reagent.

## References

[1] Hause BM, Collin EA, Peddireddi L, Yuan F, Chen Z, Hesse RA, Gauger PC, Clement T, Fang Y, Anderson G. 2015. Discovery of a novel putative atypical porcine pestivirus in pigs in the USA. J Gen Virol 96:2994–2998. 10.1099/jgv.0.000251. 26219947

[2] Gatto IRH, Arruda PH, Visek CA, Victoria JG, Patterson AR, Krull AC, Schwartz KJ, de Oliveira LG, Arruda BL. 2018. Detection of atypical porcine pestivirus in semen from commercial boar studs in the United States. Transbound Emerg Dis 65:e339–e343. 10.1111/tbed.12759. 29144025

[3] Gatto IRH, Harmon K, Bradner L, Silva P, Linhares DCL, Arruda PH, de Oliveira LG, Arruda BL. 2018. Detection of atypical porcine pestivirus in Brazil in the central nervous system of suckling piglets with congenital tremor. Transbound Emerg Dis 65:375–380. 10.1111/tbed.12824. 29393592

[4] Possatti F, Headley SA, Leme RA, Dall Agnol AM, Zotti E, de Oliveira TES, Alfieri AF, Alfieri AA. 2018. Viruses associated with congenital tremor and high lethality in piglets. Transbound Emerg Dis 65:331–337. 10.1111/tbed.12807. 29322653

[5] Mósena ACS, Weber MN, da Cruz RAS, Cibulski SP, da Silva MS, Puhl DE, Hammerschmitt ME, Takeuti KL, Driemeier D, de Barcellos DESN, Canal CW. 2018. Presence of atypical porcine pestivirus (APPV) in Brazilian pigs. Transbound Emerg Dis 65:22–26. 10.1111/tbed.12753. 29119697

[6] Chen F, Knutson TP, Braun E, Jiang Y, Rossow S, Marthaler DG. 2019. Semi-quantitative duplex RT-PCR reveals the low occurrence of Porcine Pegivirus and Atypical Porcine Pestivirus in diagnostic samples from the United States. Transbound Emerg Dis 66:1420–1425. 10.1111/tbed.13154. 30806022 PMC6849716

[7] Sutton KM, Lahmers KK, Harris SP, Wijesena HR, Mote BE, Kachman SD, Borza T, Ciobanu DC. 2019. Detection of atypical porcine pestivirus genome in newborn piglets affected by congenital tremor and high preweaning mortality1. J Anim Sci 97:4093–4100. 10.1093/jas/skz267. 31396615 PMC6776285

[8] Mósena ACS, Weber MN, Cibulski SP, Silva MS, Paim WP, Silva GS, Medeiros AA, Viana NA, Baumbach LF, Puhl DE, Silveira S, Corbellini LG, Canal CW. 2020. Survey for pestiviruses in backyard pigs in southern Brazil. J Vet Diagn Invest 32:136–141. 10.1177/1040638719896303. 31924139 PMC7003213

[9] A de G, Deijs M, Guelen L, van Grinsven L, van Os-Galdos L, Vogels W, Derks C, Cruijsen T, Geurts V, Vrijenhoek M, Suijskens J, van Doorn P, van Leengoed L, Schrier C, van der Hoek L. 2016. Atypical porcine pestivirus: a possible cause of congenital tremor type A-II in newborn piglets. Viruses 8:271. 10.3390/v8100271. PMC5086607 27782037

[10] Postel A, Hansmann F, Baechlein C, Fischer N, Alawi M, Grundhoff A, Derking S, Tenhündfeld J, Pfankuche VM, Herder V, Baumgärtner W, Wendt M, Becher P. 2016. Presence of atypical porcine pestivirus (APPV) genomes in newborn piglets correlates with congenital tremor. Sci Rep 6:27735. 10.1038/srep27735. 27292119 PMC4904412

[11] Muñoz-González S, Canturri A, Pérez-Simó M, Bohórquez JA, Rosell R, Cabezón O, Segalés J, Domingo M, Ganges L. 2017. First report of the novel atypical porcine pestivirus in Spain and a retrospective study. Transbound Emerg Dis 64:1645–1649. 10.1111/tbed.12699. 28941140

[12] Postel A, Meyer D, Cagatay GN, Feliziani F, de Mia GM, Fischer N, Grundhoff A, Milićević V, Deng M-C, Chang C-Y, Qiu H-J, Sun Y, Wendt M, Becher P. 2017. High abundance and genetic variability of atypical porcine pestivirus in pigs from Europe and Asia. Emerg Infect Dis 23:2104–2107. 10.3201/eid2312.170951. 29148382 PMC5708225

[13] Beer M, Wernike K, Dräger C, Höper D, Pohlmann A, Bergermann C, Schröder C, Klinkhammer S, Blome S, Hoffmann B. 2017. High prevalence of highly variable atypical porcine pestiviruses found in Germany. Transbound Emerg Dis 64:e22–e26. 10.1111/tbed.12532. 27297961

[14] Schwarz L, Riedel C, Högler S, Sinn LJ, Voglmayr T, Wöchtl B, Dinhopl N, Rebel-Bauder B, Weissenböck H, Ladinig A, Rümenapf T, Lamp B. 2017. Congenital infection with atypical porcine pestivirus (APPV) is associated with disease and viral persistence. Vet Res 48:1. 10.1186/s13567-016-0406-1. 28057061 PMC5217315

[15] Dénes L, Biksi I, Albert M, Szeredi L, Knapp DG, Szilasi A, Bálint Á, Balka G. 2018. Detection and phylogenetic characterization of atypical porcine pestivirus strains in Hungary. Transbound Emerg Dis 65:2039–2042. 10.1111/tbed.12981. 30105779

[16] Kaufmann C, Stalder H, Sidler X, Renzullo S, Gurtner C, Grahofer A, Schweizer M. 2019. Long-term circulation of atypical porcine pestivirus (APPV) within Switzerland. Viruses 11:653. 10.3390/v11070653. PMC6669711 31319583

[17] Sozzi E, Salogni C, Lelli D, Barbieri I, Moreno A, Alborali GL, Lavazza A. 2019. Molecular survey and phylogenetic analysis of atypical porcine pestivirus (APPV) identified in swine and wild boar from northern Italy. Viruses 11:1142. 10.3390/v11121142. PMC6950564 31835549

[18] Cagatay GN, Meyer D, Wendt M, Becher P, Postel A. 2019. Characterization of the humoral immune response induced after infection with atypical porcine pestivirus (APPV). Viruses 11:880. 10.3390/v11100880. PMC6832543 31546571

[19] Michelitsch A, Dalmann A, Wernike K, Reimann I, Beer M. 2019. Seroprevalences of newly discovered porcine estiviruses in German pig farms. Vet Sci 6:86. 10.3390/vetsci6040086. PMC6958323 31717716

[20] Yuan J, Han Z, Li J, Huang Y, Yang J, Ding H, Zhang J, Zhu M, Zhang Y, Liao J, Zhao M, Chen J. 2017. Atypical porcine pestivirus as a novel type of pestivirus in pigs in China. Front Microbiol 8:862. 10.3389/fmicb.2017.00862. 28553280 PMC5425480

[21] Zhang K, Wu K, Liu J, Ge S, Xiao Y, Shang Y, Ning Z. 2017. Identification of atypical porcine pestivirus infection in swine herds in China. Transbound Emerg Dis 64:1020–1023. 10.1111/tbed.12659. 28497656

[22] Zhang H, Wen W, Hao G, Hu Y, Chen H, Qian P, Li X. 2018. Phylogenetic and genomic characterization of a novel atypical porcine pestivirus in China. Transbound Emerg Dis 65:e202–e204. 10.1111/tbed.12675. 28710801

[23] Wu S, Wang Z, Zhang W, Deng S. 2018. Complete genome sequence of an atypical porcine pestivirus isolated from Jiangxi Province, China. Genome Announc 6:e00439-18. 10.1128/genomeA.00439-18. 29903809 PMC6003742

[24] Shen H, Liu X, Zhang P, Wang L, Liu Y, Zhang L, Liang P, Song C. 2018. Identification and characterization of atypical porcine pestivirus genomes in newborn piglets with congenital tremor in China. J Vet Sci 19:468–471. 10.4142/jvs.2018.19.3.468. 29284212 PMC5974529

[25] Pan S, Yan Y, Shi K, Wang M, Mou C, Chen Z. 2019. Molecular characterization of two novel atypical porcine pestivirus (APPV) strains from piglets with congenital tremor in China. Transbound Emerg Dis 66:35–42. 10.1111/tbed.13029. 30281923

[26] Zhou K, Yue H, Tang C, Ruan W, Zhou Q, Zhang B. 2019. Prevalence and genome characteristics of atypical porcine pestivirus in southwest China. J Gen Virol 100:84–88. 10.1099/jgv.0.001188. 30516465

[27] Zhang X, Dai R, Li Q, Zhou Q, Luo Y, Lin L, Bi Y, Chen F. 2019. Detection of three novel atypical porcine pestivirus strains in newborn piglets with congenital tremor in southern China. Infect Genet Evol 68:54–57. 10.1016/j.meegid.2018.12.008. 30529720

[28] Yin Y, Shi K, Sun W, Mo S. 2019. Complete genome sequence of an atypical porcine pestivirus strain, GX01-2018, from Guangxi Province, China. Microbiol Resour Announc 8:e01440-18. 10.1128/MRA.01440-18. 30746517 PMC6368652

[29] Xie Y, Wang X, Su D, Feng J, Wei L, Cai W, Li J, Lin S, Yan H, He D. 2019. Detection and genetic characterization of atypical porcine pestivirus in piglets with congenital tremors in southern China. Front Microbiol 10:1406. 10.3389/fmicb.2019.01406. 31281300 PMC6596314

[30] Yan XL, Li YY, He LL, Wu JL, Tang XY, Chen GH, Mai KJ, Wu RT, Li QN, Chen YH, Sun Y, Ma JY. 2019. 12 novel atypical porcine pestivirus genomes from neonatal piglets with congenital tremors: a newly emerging branch and high prevalence in China. Virology 533:50–58. 10.1016/j.virol.2019.04.010. 31103885

[31] Liu J, Li Z, Ren X, Li H, Lu R, Zhang Y, Ning Z. 2019. Viral load and histological distribution of atypical porcine pestivirus in different tissues of naturally infected piglets. Arch Virol 164:2519–2523. 10.1007/s00705-019-04345-3. 31270607

[32] Cagatay GN, Antos A, Meyer D, Maistrelli C, Keuling O, Becher P, Postel A. 2018. Frequent infection of wild boar with atypical porcine pestivirus (APPV). Transbound Emerg Dis 65:1087–1093. 10.1111/tbed.12854. 29527814

[33] Colom-Cadena A, Ganges L, Muñoz-González S, Castillo-Contreras R, Bohórquez JA, Rosell R, Segalés J, Marco I, Cabezon O. 2018. Atypical porcine pestivirus in wild boar (Sus scrofa), Spain. Vet Rec 183:569. 10.1136/vr.104824. 30201807

[34] Choe S, Park G-N, Cha RM, Hyun B-H, Park B-K, An D-J. 2020. Prevalence and genetic diversity of atypical porcine pestivirus (APPV) detected in South Korean wild boars. Viruses 12:680. 10.3390/v12060680. PMC7354535 32599836

[35] Smith DB, Meyers G, Bukh J, Gould EA, Monath T, Scott Muerhoff A, Pletnev A, Rico-Hesse R, Stapleton JT, Simmonds P, Becher P. 2017. Proposed revision to the taxonomy of the genus Pestivirus, family Flaviviridae. J Gen Virol 98:2106–2112. 10.1099/jgv.0.000873. 28786787 PMC5656787

[36] Seago J, Hilton L, Reid E, Doceul V, Jeyatheesan J, Moganeradj K, McCauley J, Charleston B, Goodbourn S. 2007. The Npro product of classical swine fever virus and bovine viral diarrhea virus uses a conserved mechanism to target interferon regulatory factor-3. J Gen Virol 88:3002–3006. 10.1099/vir.0.82934-0. 17947522

[37] Mätzener P, Magkouras I, Rümenapf T, Peterhans E, Schweizer M. 2009. The viral RNase E(rns) prevents IFN type-I triggering by pestiviral single- and double-stranded RNAs. Virus Res 140:15–23. 10.1016/j.virusres.2008.10.015. 19041350

[38] Deregt D, Loewen KG. 1995. Bovine viral diarrhea virus: biotypes and disease. Can Vet J 36:371–378. 7648541 PMC1686947

[39] Johnson DW, Muscoplat CC. 1973. Immunologic abnormalities in calves with chronic bovine viral diarrhea. Am J Vet Res 34:1139–1141. 4747034

[40] Tråvén M, Alenius S, Fossum C, Larsson B. 1991. Primary bovine viral diarrhoea virus infection in calves following direct contact with a persistently viraemic calf. Zentralbl Veterinarmed B 38:453–462. 10.1111/j.1439-0450.1991.tb00895.x. 1719713

[41] Schweizer M, Stalder H, Haslebacher A, Grisiger M, Schwermer H, Di Labio E. 2021. Eradication of bovine viral diarrhoea (BVD) in cattle in Switzerland: lessons taught by the complex biology of the virus. Front Vet Sci 8:702730. 10.3389/fvets.2021.702730. 34557540 PMC8452978

[42] Moennig V, Floegel-Niesmann G, Greiser-Wilke I. 2003. Clinical signs and epidemiology of classical swine fever: a review of new knowledge. Vet J 165:11–20. 10.1016/s1090-0233(02)00112-0. 12618065

[43] Edwards S, Fukusho A, Lefèvre P-C, Lipowski A, Pejsak Z, Roehe P, Westergaard J. 2000. Classical swine fever: the global situation. Vet Microbiol 73:103–119. 10.1016/s0378-1135(00)00138-3. 10785321

[44] Gunn GJ, Stott AW, Humphry RW. 2004. Modelling and costing BVD outbreaks in beef herds. Vet J 167:143–149. 10.1016/S1090-0233(03)00112-6. 14975388

[45] Tautz N, Tews BA, Meyers G. 2015. The molecular biology of pestiviruses. Adv Virus Res 93:47–160. 10.1016/bs.aivir.2015.03.002. 26111586

[46] Braun U, Hilbe M, Peterhans E, Schweizer M. 2019. Border disease in cattle. Vet J 246:12–20. 10.1016/j.tvjl.2019.01.006. 30902184

[47] Arruda BL, Arruda PH, Magstadt DR, Schwartz KJ, Dohlman T, Schleining JA, Patterson AR, Visek CA, Victoria JG. 2016. Identification of a divergent lineage porcine pestivirus in nursing piglets with congenital tremors and reproduction of disease following experimental inoculation. PLoS One 11:e0150104. 10.1371/journal.pone.0150104. 26909691 PMC4766193

[48] Stenberg H, Jacobson M, Malmberg M. 2020. A review of congenital tremor type A-II in piglets. Anim Health Res Rev 21:84–88. 10.1017/S146625232000002X. 32066514

[49] Chen Z, Rijnbrand R, Jangra RK, Devaraj SG, Qu L, Ma Y, Lemon SM, Li K. 2007. Ubiquitination and proteasomal degradation of interferon regulatory factor-3 induced by Npro from a cytopathic bovine viral diarrhea virus. Virology 366:277–292. 10.1016/j.virol.2007.04.023. 17531282 PMC2000802

[50] Bauhofer O, Summerfield A, Sakoda Y, Tratschin J-D, Hofmann MA, Ruggli N. 2007. Classical swine fever virus Npro interacts with interferon regulatory factor 3 and induces its proteasomal degradation. J Virol 81:3087–3096. 10.1128/JVI.02032-06. 17215286 PMC1866024

[51] Iqbal M, Poole E, Goodbourn S, McCauley JW. 2004. Role for bovine viral diarrhea virus Erns glycoprotein in the control of activation of beta interferon by double-stranded RNA. J Virol 78:136–145. 10.1128/jvi.78.1.136-145.2004. 14671095 PMC303375

[52] Magkouras I, Mätzener P, Rümenapf T, Peterhans E, Schweizer M. 2008. RNase-dependent inhibition of extracellular, but not intracellular, dsRNA-induced interferon synthesis by Erns of pestiviruses. J Gen Virol 89:2501–2506. 10.1099/vir.0.2008/003749-0. 18796719

[53] Rinck G, Birghan C, Harada T, Meyers G, Thiel HJ, Tautz N. 2001. A cellular J-domain protein modulates polyprotein processing and cytopathogenicity of a pestivirus. J Virol 75:9470–9482. 10.1128/JVI.75.19.9470-9482.2001. 11533209 PMC114514

[54] Lackner T, Müller A, König M, Thiel H-J, Tautz N. 2005. Persistence of bovine viral diarrhea virus is determined by a cellular cofactor of a viral autoprotease. J Virol 79:9746–9755. 10.1128/JVI.79.15.9746-9755.2005. 16014936 PMC1181585

[55] Wiskerchen M, Collett MS. 1991. Pestivirus gene expression: protein p80 of bovine viral diarrhea virus is a proteinase involved in polyprotein processing. Virology 184:341–350. 10.1016/0042-6822(91)90850-b. 1651596

[56] Tamura JK, Warrener P, Collett MS. 1993. RNA-stimulated NTPase activity associated with the p80 protein of the pestivirus bovine viral diarrhea virus. Virology 193:1–10. 10.1006/viro.1993.1097. 8382392

[57] Warrener P, Collett MS. 1995. Pestivirus NS3 (p80) protein possesses RNA helicase activity. J Virol 69:1720–1726. 10.1128/JVI.69.3.1720-1726.1995. 7853509 PMC188775

[58] Agapov EV, Murray CL, Frolov I, Qu L, Myers TM, Rice CM. 2004. Uncleaved NS2-3 is required for production of infectious bovine viral diarrhea virus. J Virol 78:2414–2425. 10.1128/jvi.78.5.2414-2425.2004. 14963137 PMC369244

[59] Mendez E, Ruggli N, Collett MS, Rice CM. 1998. Infectious bovine viral diarrhea virus (strain NADL) RNA from stable cDNA clones: a cellular insert determines NS3 production and viral cytopathogenicity. J Virol 72:4737–4745. 10.1128/JVI.72.6.4737-4745.1998. 9573238 PMC110005

[60] Tautz N, Thiel HJ, Dubovi EJ, Meyers G. 1994. Pathogenesis of mucosal disease: a cytopathogenic pestivirus generated by an internal deletion. J Virol 68:3289–3297. 10.1128/JVI.68.5.3289-3297.1994. 8151789 PMC236819

[61] Tautz N, Harada T, Kaiser A, Rinck G, Behrens S, Thiel HJ. 1999. Establishment and characterization of cytopathogenic and noncytopathogenic pestivirus replicons. J Virol 73:9422–9432. 10.1128/JVI.73.11.9422-9432.1999. 10516051 PMC112977

[62] Behrens SE, Grassmann CW, Thiel HJ, Meyers G, Tautz N. 1998. Characterization of an autonomous subgenomic pestivirus RNA replicon. J Virol 72:2364–2372. 10.1128/JVI.72.3.2364-2372.1998. 9499097 PMC109536

[63] Isken O, Postel A, Bruhn B, Lattwein E, Becher P, Tautz N. 2019. CRISPR/Cas9-mediated knockout of DNAJC14 verifies this chaperone as a pivotal host factor for RNA replication of pestiviruses. J Virol 93:e01714-18. 10.1128/JVI.01714-18. 30518653 PMC6384085

[64] Liu J, Ren X, Li H, Yu X, Zhao B, Liu B, Ning Z. 2020. Development of the reverse genetics system for emerging atypical porcine pestivirus using in vitro and intracellular transcription systems. Virus Res 283:197975. 10.1016/j.virusres.2020.197975. 32311384

[65] Yan M, Huang J, Chen J, Yang W, Liu G. 2020. Preparation, identification, and functional analysis of monoclonal antibodies against atypical porcine pestivirus NS3 protein. J Vet Diagn Invest 32:695–699. 10.1177/1040638720939923. 32667260 PMC7488968

[66] Cagatay GN, Antos A, Suckstorff O, Isken O, Tautz N, Becher P, Postel A. 2021. Porcine complement regulatory protein CD46 is a major receptor for atypical porcine pestivirus but not for classical swine fever virus. J Virol 95:e02186-20. 10.1128/JVI.02186-20. 33568504 PMC8104093

[67] Gilmartin AA, Lamp B, Rümenapf T, Persson MAA, Rey FA, Krey T. 2012. High-level secretion of recombinant monomeric murine and human single-chain Fv antibodies from Drosophila S2 cells. Protein Eng Des Sel 25:59–66. 10.1093/protein/gzr058. 22160929 PMC3258843

[68] Yi Z, Yuan Z, Rice CM, MacDonald MR. 2012. Flavivirus replication complex assembly revealed by DNAJC14 functional mapping. J Virol 86:11815–11832. 10.1128/JVI.01022-12. 22915803 PMC3486285

[69] Kasza L, Shadduck JA, Christofinis GJ. 1972. Establishment, viral susceptibility and biological characteristics of a swine kidney cell line SK-6. Res Vet Sci 13:46–51. 10.1016/S0034-5288(18)34087-6. 4336054

[70] Corapi WV, Donis RO, Dubovi EJ. 1988. Monoclonal antibody analyses of cytopathic and noncytopathic viruses from fatal bovine viral diarrhea virus infections. J Virol 62:2823–2827. 10.1128/JVI.62.8.2823-2827.1988. 2455820 PMC253717

[71] Gallei A, Blome S, Gilgenbach S, Tautz N, Moennig V, Becher P. 2008. Cytopathogenicity of classical swine fever virus correlates with attenuation in the natural host. J Virol 82:9717–9729. 10.1128/JVI.00782-08. 18653456 PMC2546992

[72] Kiesler A, Plankensteiner J, Schwarz L, Riedel C, Seitz K, Mötz M, Ladinig A, Lamp B, Rümenapf T. 2021. Prevalence of linda virus neutralizing antibodies in the Austrian pig population. Viruses 13:1001. 10.3390/v13061001. 34071946 PMC8229103

[73] Lamp B, Riedel C, Roman-Sosa G, Heimann M, Jacobi S, Becher P, Thiel H-J, Rümenapf T. 2011. Biosynthesis of classical swine fever virus nonstructural proteins. J Virol 85:3607–3620. 10.1128/JVI.02206-10. 21270154 PMC3067844

[74] Reuscher CM, Schmidt L, Netsch A, Lamp B. 2021. Characterization of a cytopathogenic reporter CSFV. Viruses 13:1209. 10.3390/v13071209. 34201706 PMC8310069

[75] Becher P, Orlich M, Thiel HJ. 1998. Ribosomal S27a coding sequences upstream of ubiquitin coding sequences in the genome of a pestivirus. J Virol 72:8697–8704. 10.1128/JVI.72.11.8697-8704.1998. 9765411 PMC110283

